# Integrin α3β1 promotes vessel formation of glioblastoma-associated endothelial cells through calcium-mediated macropinocytosis and lysosomal exocytosis

**DOI:** 10.1038/s41467-022-31981-2

**Published:** 2022-07-25

**Authors:** Eunnyung Bae, Ping Huang, Gaёlle Müller-Greven, Dolores Hambardzumyan, Andrew Edward Sloan, Amy S. Nowacki, Nicholas Marko, Cathleen R. Carlin, Candece L. Gladson

**Affiliations:** 1 Department of Cancer Biology, Cleveland, Clinic, Cleveland, OH USA; 2 Departments of Oncological Sciences and Neurosurgery, Icahn School of Medicine, Mount Sinai, New York, NY USA; 3 Department of Neurosurgery, Seidman Cancer Center, Cleveland, OH USA; 4 University Hospital-Cleveland Medical Center and the Case Comprehensive Cancer Center, Case Western Reserve University, School of Medicine, Cleveland, OH USA; 5 Department of Quantitative Health Sciences, Cleveland Clinic, Cleveland, OH USA; 6 Department of Neurosurgery, LewisGale Medical Center, Salem, VA USA; 7 Department of Molecular Biology and Microbiology, Case Western Reserve University, School of Medicine, Cleveland, OH USA; 8 The Brain Tumor and Neuro-Oncology Center, Cleveland Clinic, Cleveland, OH USA

**Keywords:** Tumour angiogenesis, Molecular medicine

## Abstract

Therapeutic targeting of angiogenesis in glioblastoma has yielded mixed outcomes. Investigation of tumor-associated angiogenesis has focused on the factors that stimulate the sprouting, migration, and hyperproliferation of the endothelial cells. However, little is known regarding the processes underlying the formation of the tumor-associated vessels. To address this issue, we investigated vessel formation in CD31^+^ cells isolated from human glioblastoma tumors. The results indicate that overexpression of integrin α3β1 plays a central role in the promotion of tube formation in the tumor-associated endothelial cells in glioblastoma. Blocking α3β1 function reduced sprout and tube formation in the tumor-associated endothelial cells and vessel density in organotypic cultures of glioblastoma. The data further suggest a mechanistic model in which integrin α3β1-promoted calcium influx stimulates macropinocytosis and directed maturation of the macropinosomes in a manner that promotes lysosomal exocytosis during nascent lumen formation. Altogether, our data indicate that integrin α3β1 may be a therapeutic target on the glioblastoma vasculature.

## Abstract (web summary)

Tumour-associated angiogenesis facilitates the growth of tumours. Here the authors show that integrin α3β1 promotes blood vessel formation in glioblastoma through calcium-mediated macropinocytosis and lysosomal exocytosis.

## Introduction

Glioblastoma (GBM) is the most common primary brain tumor and most malignant form of glioma^1^. Despite current standard-of-care therapy involving surgical resection followed by radiation with concomitant temozolomide, the median overall survival is approximately 15 months^1,2^. As tumor necrosis and microvascular hyperplasia are pathological hallmarks of GBM^1^, the tumor vasculature in GBM is considered a prime target for drug development^2^.

Normal brain vasculature is composed of endothelial cells (ECs), pericytes, and astrocytes. These cells form the blood-brain barrier that selectively restricts entry of molecules into the intracerebral circulatory system^3^. Typically, vessels in GBM tumors form glomeruloid bodies with multiple layers of ECs and pericytes, and a thickened and duplicated basement membrane. These vessels are not only morphologically abnormal but also dysfunctionally, with a high level of permeability that contributes to a compromised blood brain barrier^1,3^.

New vessel formation from the existing vasculature occurs through angiogenesis^4^. Angiogenesis is a complex process that requires proliferation, migration, and alignment of the ECs in a manner that results in formation of vessels with a patent lumen. It is initiated by the development of sprouts from the existing vasculature. Sprout development starts with formation of a tip cell in response to angiogenic stimuli, such as vascular endothelial growth factor (VEGF). The tip cell migrates toward angiogenic stimuli-forming sprouts, and stalk cells follow the tip cells to extend and fuse the sprouts. Lumen formation occurs simultaneously as a part of the associated tubulogenesis process^4^. ECs interact extensively with the extracellular matrix. Non-brain ECs have been shown to exhibit pinocytosis (commonly termed macropinocytosis), which mediates non-selective uptake from the extracellular environment by actin-dependent membrane ruffles that fuse back with the plasma membrane to generate large endocytic vacuoles called pinosomes (or macropinosomes)^5,6^. In certain 3D matrices, these non-brain ECs undergo macropinocytosis at the basal membrane that is associated with the extracellular matrix, followed by directed trafficking of the macropinosomes to the apical surface where they fuse with the cell membrane during nascent lumen formation^5^.

Integrins, a family of heterodimeric receptors, have been shown to play critical roles in orchestrating angiogenesis through multiple diverse mechanisms. Integrin functions are mediated by interactions with the extracellular matrix and the cytoskeleton that have been implicated in every step of EC tubulogenesis, including cell migration, proliferation, cytoskeletal reorganization controlling cell–cell interactions and cell polarity, and cross-talk with angiogenesis-stimulating growth factor receptors^5,7–9^.

Increased tumor-associated angiogenesis is widely accepted as a mechanism that supports the growth of tumors^4,7^. To date, several integrins, including αvβ3, αvβ5 and α5β1, have been shown to play key roles in tumor-associated angiogenesis^7,8^, and integrin α3β1 has been implicated in tumor-associated increases in angiogenesis^10–12^. Cilengitide, a cyclic RGD peptide that targets αv integrins, has been tested in clinical trials as a single agent or in combination with radio-chemo-therapy for GBM with mixed outcomes^2,13^. Similarly, because of the vital role of VEGF in stimulating angiogenesis, an anti-VEGF humanized monoclonal antibody (bevacizumab) and small molecule tyrosine kinase inhibitors targeting VEGF receptors (VEGFR) have been developed and administered as anti-angiogenic therapy for GBM^2,14^. However, in spite of benefits such as reduced edema and transient vascular normalization, no overall survival advantage has been reported and resistance frequently develops^2,14^.

The mechanisms responsible for the development of resistance to current anti-angiogenic therapies and the mixed outcomes in GBM are still being elucidated. A contributing factor could be unconventional mechanisms of neovascularization, such as trans-differentiation of GBM cancer stem cells to ECs and pericytes^15–17^. While lumen and tube formation in normal non-brain ECs has been studied extensively in vitro and in vivo using animal models^5,18–23^, to our knowledge the mechanisms underlying lumen and tube formation in endothelial cells in GBM or other tumors have not been investigated. An improved understanding of the mechanisms by which tumor-associated ECs or EC-like heterogenous cells form the neovasculature in GBM could form the basis for optimization of therapeutic targeting of the vasculature in GBM. We therefore investigated the tube formation process of tumor-associated CD31^+^ cells isolated from GBM tumors (TECs) and CD31^+^ cells isolated from normal brain (NECs). Collectively, our data show that integrin α3β1 is upregulated in TECs, and both tube formation and sprouting by TECs can be targeted by functional blockade of integrin α3β1 in vitro and ex vivo. They further suggest a mechanistic model in which (i) elevated macropinocytosis in TECs is associated with tubulogenesis through directed maturation of macropinosomes to lysosomes supporting development of the nascent lumen through exocytosis, and (ii) integrin α3β1 mediated upregulation of vessel formation in TECs through enhanced calcium influx that drives the increased macropinocytosis, lysosomal exocytosis and lumen formation. These data indicate that integrin α3β1 has a central role in blood vessel formation in GBM.

## Results

### TECs show more rapid tube formation and greater sprout generation relative to NECs

**Fig. 1:**
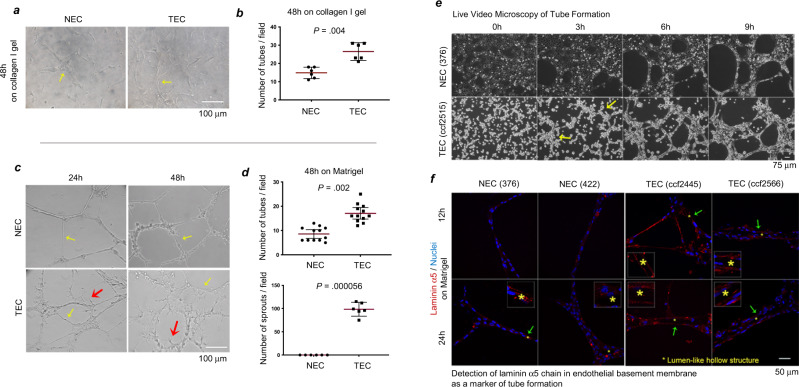
Elevated numbers of tubes and sprouts formed by TECs as compared to NECs and more rapid tube formation in TECs. **a**, **b** NECs (422) and TECs (isolate ccf2687) were cultured for 48 h in collagen gels as described in Methods. Representative images are shown in **a**, **b** is the corresponding statistical analysis by two-sided Wilcoxon rank-sum tests. Yellow arrows denote tubes. *n* = 6 different fields in each group. **c**, **d** NECs (623) and TECs (isolate ccf2445) were cultured on Matrigel for 24 h or 48 h. Representative images are shown in **c**, **d** is the corresponding statistical analysis by two-sided Wilcoxon rank-sum tests. Yellow arrows denote tubes and red arrows denote sprouts. For tube numbers, *n* = 12 different fields in each group; and for sprout numbers, *n* = 6 different fields in each group. Red and black bars in **b**, **d** indicate means with 95% confidence intervals. **e** Live video microscopy of NECs (376) and TECs (isolate ccf2515) over the first 9 h of culture on Matrigel. Representative images are shown. **f** Immunofluorescent staining of laminin α5 chain (sc-16592; Alexa-Fluor-594; red) in the basement membrane of tubes formed by NECs (376 and 422) and TECs (isolates ccf2445 and ccf2566) on Matrigel for 12 and 24 h. Cell nuclei stained with DAPI (blue). Independent experiments were performed at least two times with similar results. Source data are provided as a source data file.

We first investigated whether TECs isolated from human GBM tumors^24^ differ from NECs in the kinetics of tube formation and generation of sprouts using an in vitro model, in which TEC and NEC isolates were cultured in collagen gels or on Matrigel. TECs formed higher numbers of tubes/field than NECs both in collagen gels (Fig. 1a, b) and on Matrigel (Fig. 1c, d). During the time frame of these experiments, sprouts were only observed on the TECs (Fig. 1c, d, red arrows). In live video microscopy of tube formation on Matrigel (starting at 0 h post-seeding), tube-like structures were observed earlier in TECs as compared to NECs (Fig. 1e and Supplementary Fig. 1), suggesting more rapid tube formation by TECs. The higher number of tubes and sprout formation in the TECs is not due to sorting for CD31 during the isolation of TECs, as sorting of NECs for CD31 had no effect on tube formation (Supplementary Fig. 2). The laminin-α5 chain is involved in EC tube formation^25^ and is typically found in the basement membrane of blood vessel walls. We therefore used immunofluorescence analysis of the laminin-α5 chain to determine if the tube-like structures formed lumens. We observed immunofluorescent staining of the laminin-α5 chain around lumen-like hollow structures in confocal images of TECs at an earlier time point (12 h) than those found in the NECs (24 h) (Fig. 1f). Moreover, the intensity of the staining of the laminin-α5 chain was stronger in the TECs (Fig. 1f).

### Higher expression of the integrin α3 subunit in TECs isolated from human GBM

**Fig. 2:**
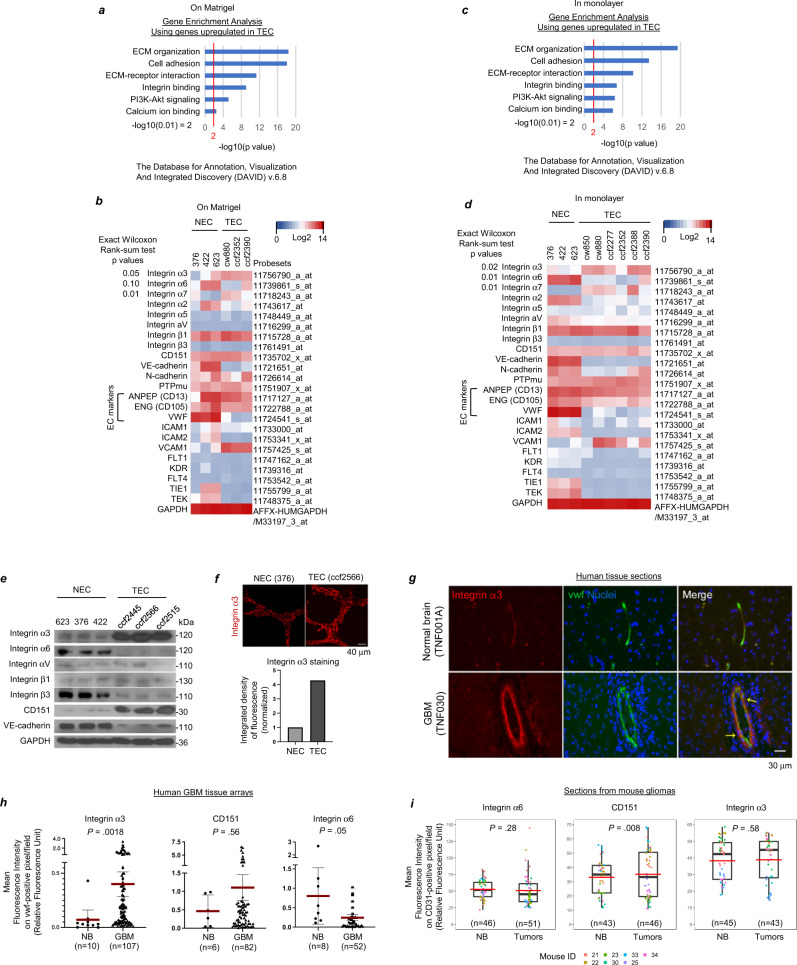
Expression of the integrin α3 subunit is elevated in TECs from human glioblastoma compared to NECs. **a**, **b** Total RNA was extracted from 3 NECs (376, 422, and 623) and 3 TECs (isolates cw880, ccf2352, and ccf2390) when similar densities of tubes had formed on Matrigel, and **c**, **d** from monolayers of 6 TECs (cw850, cw880, ccf2277, ccf2352, ccf2388, and ccf2390) and 3 NECs (375, 422, and 623), followed by gene expression microarray (HG-U219). **a**, **c** Genes upregulated over 2-fold in TECs compared to NECs were subject to gene set enrichment and pathway analysis using DAVID Bioinformatics Resources 6.8 (https://david.ncifcrf.gov), where *p*-values were calculated by Fisher’s exact test. **b**, **d** Expression profiles of integrins and genes relevant to angiogenesis both on Matrigel and in monolayer are shown as heatmaps; *p*-values were obtained by two-sided exact-Wilcoxon-rank-sum tests, without adjustment for multiple comparisons. **e** Cell lysates derived from the same experiment with monolayers of NECs (623, 376, and 422) and TECs (ccf2445, ccf2566, and ccf2515) were subjected to western blot analysis with the indicated antibodies in parallel. **f** Representative confocal images of integrin α3 staining of tubes formed by NECs (376) or TECs (ccf2566) plated on Matrigel (24 h). Graph represents the integrated densities of integrin α3-immunofluorescence. **g** Paraffin sections of human GBM and normal brain (NB) samples were double-labeled for integrin α3 and vwf, followed by DAPI staining. Representative images shown. Colocalization of the integrin α3 signal with the vwf signal for GBM-sections was 86%, based on the Manders’ Coefficient (M1 = 0.863; M2 = 0.515). **h** Human GBM tissue arrays containing NB were double-labeled for vwf and integrin α3, CD151, or integrin α6. Statistical analysis: two-sided Wilcoxon-rank-sum-tests; red lines denote means and black lines denote 95% confidence intervals. **i** Tumor sections from mouse glioma were double-labeled by immunofluorescence for integrin α3, CD151, or integrin α6 and CD31. Average fluorescence intensities of integrin α3, CD151, or integrin α6 on CD31-positive pixels/field were obtained from tumor or adjacent NB using ImageJ and represented as boxplots. Boxes indicate first and third quartiles, bands indicate medians, and whiskers indicate ±1.5 interquartile range. Statistical analysis: linear mixed model; red bars, means. (**h**, **i**) On the *x* axis, *n* = number of different fields. **e**–**g** Independent experiments were performed at least two times with similar results. Source data are provided as a source file.

To compare gene expression, three different NEC isolates and three different TEC isolates plated on Matrigel were harvested when similar densities of tubes were observed as determined by microscopic examination (Fig. 2a, b). Three different NEC isolates and six different TEC isolates in monolayer plated on gelatin were also harvested (Fig. 2c, d). Total RNA was extracted and assessed using gene expression microarrays (Affymetrix, HG-U219). We found that the gene expression patterns were different between TECs and NECs, while they were similar within each group. Prior studies of differential gene expression between TECs and NECs have reported increased expression of TEM7 (PLXDC1), IGFBP7, SPARC, IL-8, and RDC1 (ACKR3/CXCR7) in TECs in GBM^26–29^. We did not find over- expression of these genes in our study (Supplementary Fig. 3), likely due to the differences in experimental approaches.

Gene set enrichment and pathway analyses^30–32^, performed on the subset of genes that were >2 fold-upregulated in the TEC-group as compared to the NEC-group, showed enrichment in multiple pathways. These included pathways activated by extracellular matrix-receptor interactions and integrin binding, the phosphoinositide-3-kinase (PI3k)-Akt signaling pathway, and pathways induced by calcium ion binding (Fig. 2a, c). On analyzing the gene expression of integrins and other genes relevant to angiogenesis in the gene expression microarrays, we found that integrin α3 was overexpressed in TECs, whereas integrin α6 and vascular endothelial (VE)-cadherin were under expressed as compared to NECs (Fig. 2b, d). There was no difference in the expression of integrin β1, which forms heterodimers with integrin α3 and multiple other integrin α subunits. Due to the highly similar differences in gene expression found between NECs and TECs when plated on Matrigel or when plated in monolayer conditions, we combined the array data for the TECs in the two conditions and the array data for the NECs in the two conditions, and adjusted for the plating medium when exploring each gene with a multivariable linear regression model. We then applied the Benjamini-Hochberg Procedure with a False Discovery Rate of 10% to address the issue of multiple testing. We found a significant difference in gene expression for integrin α3 (*p* = 0.0027), integrin α6 (*p* = 0.0004) and integrin α7 (*p* = 0.0007) between the NECs and TECs. Upon validation using real-time qRT-PCR and immunoblotting, the levels of integrin α3 subunit mRNA were found to be five-fold higher (Fig. S4), and the levels of the integrin α3 subunit protein seven-fold higher (Fig. 2e), in the TECs versus the NECs.

A tetraspanin, CD151, associates with laminin-binding integrins including integrin α3β1, α6β1, and α6β4^33^. CD151 has been shown to associate with integrin α3β1 through an extracellular domain of integrin α3^34^. Even though we did not find a significant difference in CD151 expression between the TEC- and NEC-groups in our microarrays, it was upregulated at both the mRNA and protein level in the TECs (Fig. 2e and Supplementary Fig. 4).

### Higher expression of the integrin α3 subunit in TECs in human GBM biopsies relative to NECs in normal brain in vivo

On examining protein expression during tube formation by immunofluorescent staining, we found strong staining for the integrin α3 subunit on tubes formed by TECs plated on Matrigel (Fig. 2f), whereas VE-cadherin staining was minimal (Supplementary Fig. 5). Similarly, double-label immunofluorescence analysis for integrin α3 and the EC marker, von Willebrand factor (vwf), on tissue obtained from the Cleveland Clinic Brain Tumor Bank and the Department of Pathology showed significantly higher mean fluorescence intensity of the integrin α3 subunit in TECs in eight GBM as compared to NECs in nine normal brains (Fig. 2g and Supplementary Fig. 6). On double-label analysis of human GBM tissue arrays (US Biomax, Inc., GL805a, GL805b and BS17017a) for the integrin α3 subunit and vwf, we found the mean fluorescence intensity of the integrin α3 subunit on vwf-positive pixels was significantly higher in GBM tumors as compared to normal brain (Fig. 2h).

We also analyzed tissue sections from mouse glioma using double-label-immunofluorescence for mouse CD31 and the mouse integrin α3 subunit, integrin α6 subunit, or CD151. The mouse model of high-grade glioma used, which displays characteristics of human GBM, was created through intracerebral injection of vector-infected chicken fibroblasts (DF-1) producing RCAS-PDGF-B virus into Nestin*-tva, Ink4a-arf* −/− transgenic mice^35^. We found the mean fluorescence intensity of the integrin α3 subunit on CD31-positive pixels/field was not significantly higher in tumor sections from the mouse model, as compared to the adjacent normal brain (Fig. 2i). However, the mean fluorescence intensity of CD151 on CD31-positive pixels/field was higher, which is consistent with the previously reported key role of CD151 in pathogenic angiogenesis in the mouse system^36^.

Collectively, our results generated using gene expression microarrays, validation studies using isolated TECs and NECs, and immunofluorescence analysis of human GBM tumors and normal brain as well as GBM tissue arrays, provide strong evidence that expression of the integrin α3 subunit is increased in TECs of human GBM tumors in vivo.

### Functional block of integrin α3β1 decreases tube formation of TECs

**Fig. 3:**
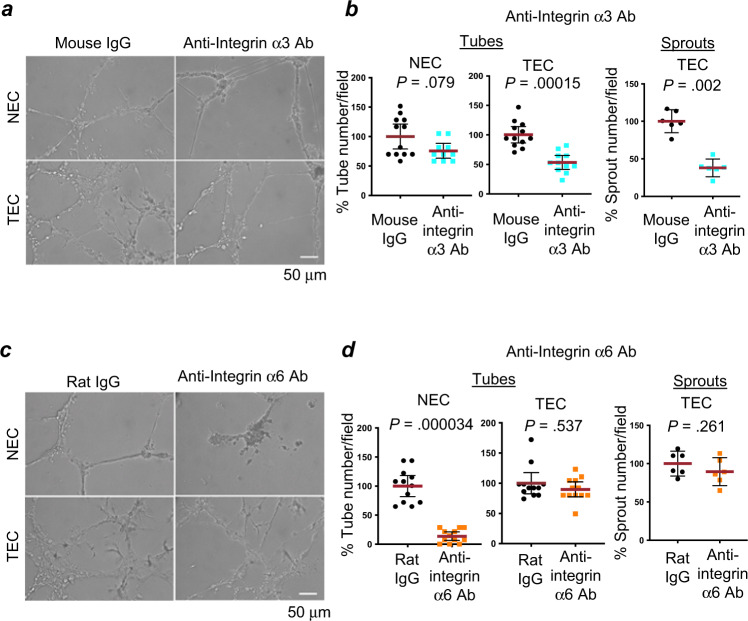
A function blocking antibody directed towards integrin α3β1 significantly reduces tube and sprout formation by TECs. **a**, **c** NECs (623) or TECs (isolate ccf2445) were incubated with function blocking antibodies towards the integrin α3 subunit or integrin α6 subunit, or control IgG (mouse IgG or rat IgG) at 5 µg/ml when plated on growth factor-reduced Matrigel in M199 medium +10% FBS for 43 h. Representative images are shown. **b**, **d** The mean numbers of tubes or sprouts in the mouse IgG or rat IgG-treated group are represented as 100%. Dot plots show % mean of the numbers of tubes or sprouts (red lines) and the 95% confidence interval (black lines). Statistical analyses: two-sided Wilcoxon-rank-sum tests. Independent experiments were performed at least two times with similar results. *n* denotes the number of different fields. **b** The *n* for tubes: NECs, *n* = 12 for mouse IgG and *n* = 10 for anti-integrin α3; and TECs, *n* = 12 for mouse IgG and *n* = 11 for anti-integrin α3. The *n* for sprouts: TECs, *n* = 6 for mouse IgG and *n* = 6 for anti-integrin α3. **d** The *n* for tubes: NECs, *n* = 12 for rat IgG and *n* = 12 for anti-integrin α6; and TECs, *n* = 12 for rat IgG and *n* = 12 for anti-integrin α6. The *n* for sprouts: TECs, *n* = 6 for rat IgG and *n* = 6 for anti-integrin α6. Source data are provided as a source data file.

Based on the gene expression microarrays and validation studies (Fig. 2 and Supplementary Figs. 4, 5, and 6), we utilized TECs and NECs plated on Matrigel for further experimental analyses of TEC function. To investigate the potential role of up-regulated integrin α3β1 expression in tube formation of TECs, we incubated TECs or NECs with a function-blocking antibody specific for the integrin α3 subunit (P1B5). This antibody recognizes the epitope sequence NTVKN, which is located at the boundary between the N-terminal repeats 1 and 2^37^ and is part of the β-propeller structure of the integrin α3 subunit that is responsible for laminin-binding by the α3β1 heterodimer^33^. Matrigel, which is a basement membrane extract from the Engelbreth-Holm-Swarm mouse sarcoma, is composed of ~60% laminin. As laminin is a ligand for both integrins α3β1 and α6β1, control experiments were carried out in parallel using a function blocking antibody specific for the integrin α6 subunit (GoH3). Treatment of TECs with P1B5 resulted in a 40% decrease in the mean number of tubes, and a 62% decrease in the mean number of sprouts, whereas treatment of NECs with P1B5 had no significant effect on the number of tubes (Fig. 3a, b). In contrast, treatment with GoH3 resulted in an 86% decrease in the mean number of tubes in the NECs, but had no significant effect on the number of tubes or sprouts in the TECs (Fig. 3c, d). A function blocking antibody to the integrin α7 subunit (integrin α7β1 is another laminin receptor) significantly reduced tube formation of the TECs but did not reduce sprout formation (Supplementary Fig. 7), indicating that integrin α7β1 also plays a role in tube formation of TECs. These data taken together suggest that, when the cells are plated on Matrigel, integrin α3β1 plays a major role in both tube and sprout formation by TECs. In contrast, α3β1 was dispensable for tube formation in NECs, where the alternative laminin receptor (integrin α6β1) is likely to have a prominent role.

### Functional block of integrin α3β1 decreases blood vessel density in organotypic cultures of GBM

**Fig. 4:**
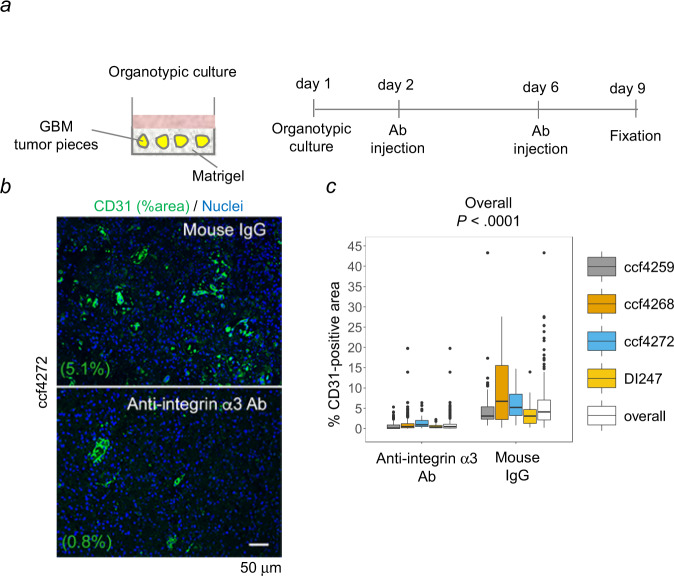
A function blocking antibody directed towards integrin α3β1 reduces blood vessel density in GBM tumors in organotypic culture. **a** Schematic diagram of the experimental procedure. Briefly, GBM tumors (ccf4259, ccf4268, ccf4272, and DI247) cut into ~4 mm in diameter were cultured in Matrigel in neurobasal medium supplemented with 1 ng/ml EGF and 1 ng/ml bFGF. On day 2, anti-integrin α3 subunit blocking antibody or control mouse IgG was injected at three places per tumor piece to achieve ~5 µg/ml in tumor volume. The same injection was repeated on day 6. On day 9, tumors were fixed in 10% formalin and paraffin-embedded. Sections were labeled with rabbit anti-CD31 antibody and Alexa-Fluor-488 goat anti-rabbit IgG, followed by DAPI nuclear stain and imaging. **b** Representative immunofluorescent images from organotypic culture of tumor ccf4272 are shown. **c** Percent CD31-positive area per field was obtained by image processing using ImageJ. *n* denotes the number of different fields, for ccf4269, *n* = 44 for mouse IgG and *n* = 95 for anti-integrin α3; for ccf4268, *n* = 39 for mouse IgG and *n* = 103 for anti-integrin α3; for ccf4272, *n* = 57 for mouse IgG and *n* = 82 for anti-integrin α3; for DI-247, *n* = 59 for mouse IgG and *n* = 88 for anti-integrin α3. The boxes indicate the first and third quartiles, bands indicate medians, and the whiskers indicate ±1.5 interquartile range. A linear mixed model was used for statistical analysis (*p* < 0.001). Source data are provided as a source data file.

To determine whether our results were reflective of the in vivo responses in the tumor microenvironment, we incubated human GBM tumor in organotypic culture with P1B5. Active capillary forming ability and good survival of ECs has been reported in organotypic culture of rat brain slices^38^. A 3D-embedded approach was used, in which human GBM tumor pieces (4 mm-diameter; ~33 mm^3^ in volume) from four patients were embedded in Matrigel. These cultures were subsequently injected on days 2 and 6 with either P1B5 or control mouse IgG, to achieve a final concentration of ~5 µg/ml per tumor piece (Fig. 4a). On day 9, the Matrigel was fixed in formalin, embedded in paraffin, and sections subjected to immunofluorescence analysis for an EC marker, CD31. In all four GBM tumors, the percent CD31-positive area was significantly lower in the tumors treated with P1B5 as compared to tumors treated with control mouse IgG (Fig. 4b, c). Collectively, our in vitro studies of TECs and NECs on Matrigel, and our ex vivo studies of GBM tumors in organotypic culture, suggest that blockade of integrin α3β1 specifically targets tumor-derived blood vessels, with minimal effect on normal blood vessels.

### Macropinocytosis is enhanced in GBM TECs and is promoted by integrin α3β1/CD151

**Fig. 5:**
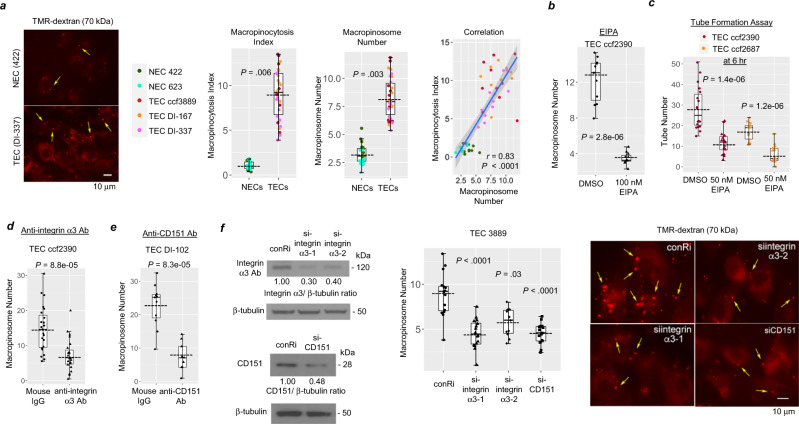
Macropinocytosis promoted by integrin α3β1/CD151 is higher in TECs and inhibition of macropinocytosis reduces tube formation. **a** NECs (422 and 623) and TECs (isolates ccf3889, DI-167, and DI-337) were incubated with 25 µg/ml TMR-dex for 30 min, followed by imaging. *n* denotes the number of different fields: NECs, *n* = 20 for 422 and *n* = 20 for 623; and TECs, *n* = 30 for each isolate. Representative images are shown. Macropinocytosis Index was defined as (total TMR-dex area/total cell area) x 100 per field and Macropinosome Number was defined as the average number of macropinosomes in a cell per field. Statistical analysis: two-sided Wilcoxon-rank-sum tests. Correlation between Macropinocytosis Index and Macropinosome Number was analyzed by linear regression model after adjusting for group effect; shaded area in the correlation graph represents the 95% confidence band for the regression line. **b** TECs (isolate ccf2390) treated with DMSO (vehicle-control; *n* = 11) or 100 nM EIPA (*n* = 11) for 10 min were subsequently incubated with TMR-dex for 30 min, followed by imaging. Statistical analysis: two-sided Wilcoxon-rank-sum test. **c** Tube formation of TECs (isolates cf2390 and ccf2687) was quantitated by the numbers of tubes formed per field on Matrigel after 6 h in M199 + 10% FBS in the presence of DMSO (vehicle-control; *n* = 18 for ccf2390, and *n* = 18 for ccf2687) or 50 nM EIPA (*n* = 18 for ccf2390, and *n* = 18 for ccf2687). Statistical analysis: two-sided Wilcoxon-rank-sum test. **d**, **e** TECs (isolate ccf2390) were pretreated for 3 h with blocking antibody toward integrin α3 (*n* = 23) or mouse IgG (*n* = 23) and TECs (isolate DI-102) were pretreated with blocking antibody toward CD151 (*n* = 11) or mouse IgG (*n* = 9), and then TECs were incubated for 30 min with TMR-dex. Statistical analysis: two-sided Wilcoxon-rank-sum test. **f** Shown are immunoblots of integrin α3 and CD151 from TECs (isolate ccf3889) at 48 h after transfection with siRNA for integrin α3 or CD151. These immunoblots were performed at least two times, and cell lysate samples loaded in separate lanes were from independent replicates. At 48 h after transfection with siRNA, TECs (isolate ccf3889) were incubated with TMR-dex for 15 min. *n* = number of different fields: integrin α3, *n* = 16 for siintegrin α3-1 and *n* = 16 for siintegrin α3-2; CD151, *n* = 17 for siCD151; and control siRNA, *n* = 16. Representative images are shown. Statistical analysis: Dunn-method for pairwise-comparisons between each siRNA with ConRi. Boxes indicate the first and third quartiles, bands indicate medians, and whiskers indicate ±1.5 interquartile range. Dotted lines denote means in **a**–**f**. Source data are provided as a source data file.

Macropinocytosis has been implicated in tube formation in non-brain ECs^5^. On measurement of the efficiency of uptake of tetramethylrhodamine-conjugated 70 kDa-dextran (TMR-dex) after a short period of incubation^39,40^, we observed significantly greater uptake in TECs as compared to NECs (Fig. 5a). Macropinocytosis Index was defined as (total TMR-dex area/total cell area) × 100 per field^40^ and Macropinosome Number as the average number of macropinosomes in a cell per field^41^. To confirm that the uptake of the TMR-dex in TECs was mediated by macropinocytosis, we used two approaches. First, immunofluorescent staining for sorting nexin 5 (SNX5), which associates with newly-formed macropinosomes^6^, indicated that many vesicles containing TMR-dex were positive for SNX5 (Supplementary Fig. 9). Second, treatment with an inhibitor of Na/H + exchange, 5-(N-ethyl-N-isopropyl) amiloride (EIPA), which inhibits macropinocytosis by preventing the Rac1 and Cdc42-mediated signaling that is required for actin polymerization^42^, reduced the uptake of TMR-dex in TECs by 63% (Fig. 5b and Supplementary Fig. 14), without a significant increase in cytotoxicity at the concentration (50 and 100 nM) used to inhibit macropinocytosis (Supplementary Figs. 10 and 14). The enhanced macropinocytosis in TECs relative to NECs was not due to CD31 sorting during the isolation of TECs, as macropinocytosis in NECs after sorting for CD31 was unchanged (Supplementary Fig. 8). Notably, the treatment with EIPA not only reduced macropinocytosis in the TECs, but also resulted in a significant decrease in tube formation (Fig. 5c).

To determine if the upregulation of integrin α3β1/CD151 pathway in GBM TECs is involved in the enhanced macropinocytosis, we used function blocking antibody and small interfering RNA (siRNA) approaches. Treatment with function blocking antibody P1B5 (directed toward the integrin α3 subunit) decreased the mean macropinosome number by 49%, and treatment with the function blocking antibody 1A5 (directed toward CD151)^43^ decreased the percent of TMR-dex-positive TECs by 65% (Fig. 5d, e). Representative images of macropinocytosis after treatment with EIPA or blocking antibodies are shown in Supplementary Fig. 11. Transfection of TECs with siRNA targeting either the integrin α3 subunit or CD151 decreased the number of TMR-dex-positive macropinosomes per cell by nearly 50% (Fig. 5f). These results suggest that integrin α3β1/CD151 promotes macropinocytosis in TECs.

Macropinocytosis can act to increase the uptake of extracellular fluid and nutrients that promote mTORC1 activity and enhance metabolic activity in general^44^. Consistent with this we observed higher mTORC1 and Akt activity in TECs (Supplementary Fig. 12a).

### Lumen formation in TECs is promoted by maturation of macropinosomes to lysosomes and lysosomal exocytosis

The observation that treatment with EIPA significantly decreased tube formation of TECs (Fig. 5c) is consistent with the concept of a specific link between macropinocytic activity and tube formation. Prior studies in non-brain ECs have suggested that coalescence of cytoplasmic vacuoles initially formed in individual ECs subsequently span adjacent ECs. EC membranes then surround the coalesced vacuoles thereby generating a new lumen^5^. This model of vessel formation is supported by studies in the zebrafish^20,22^.

**Fig. 6:**
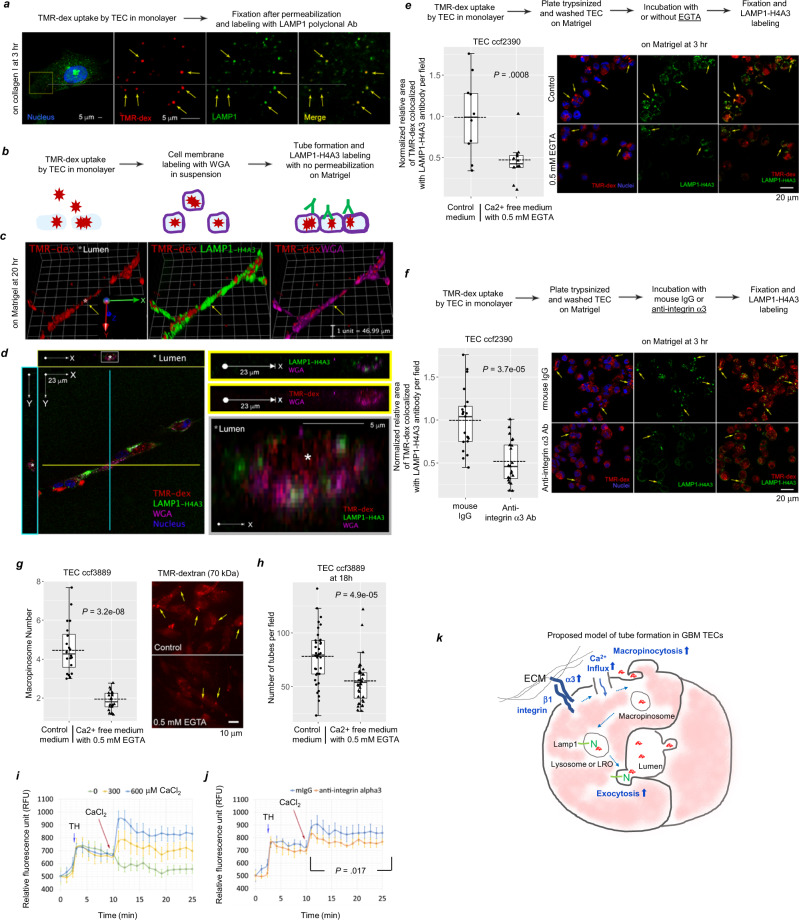
Ca^2+^-mediated macropinocytosis and lysosomal exocytosis are part of the tube formation process, and are enhanced by integrin α3β1 through Ca^2+^-influx. **a** LAMP1 was labelled after TECs (isolate ccf2390) were incubated with 25 µg/ml TMR-dex for 3 h at 37 °C, followed by fixation and permeabilization. Representative images shown; yellow arrows indicate TMR-dex and LAMP1 co-localization. Colocalization was 99% based on the Manders’ Coefficient (M1 = 0.999; M2 = 0.058). Independent experiments were performed at least two-times with similar results. **b** Experimental-procedure schematic for **c**, **d**. Adherent TECs (isolate ccf3889) were incubated with 100 µg/ml TMR-dex for 15 min at 37 °C, washed, harvested, labeled with 5 µg/ml Alexa-Fluor-647-conjugated-WGA in suspension, washed and plated on Matrigel for 20 h. Representative 3-D (**c**) and cross-sectional (**d**) images of tubes. **e**, **f** Adherent TECs (isolate ccf2390) were incubated with 100 µg/ml TMR-dex for 15 min at 37 °C, washed, harvested, and plated on Matrigel for 3 h with (*n* = 12) or without 0.5 mM EGTA (*n* = 10) (**e**), or in the presence of mouse IgG (*n* = 21) or integrin α3 antibody (*n* = 21) (**f**), followed by fixation and labeling with the LAMP1 mAb, H4A3. **g** TECs (isolate ccf3889) either in DMEM + 5% FBS (control media) or in calcium-free media were treated with (*n* = 20) or without 0.5 mM EGTA (*n* = 20) for 15 min, and then incubated with 100 µg/ml TMR-dex for 15 min at 37 °C, and fixed. Representative images are shown for panels **e**–**g**. **h** TECs (isolate ccf3889) in DMEM + 5% FBS were incubated on Matrigel with (*n* = 36) or without 0.5 mM EGTA (*n* = 36) for 18 h. **g**, **h** Statistical analysis: two-sided Wilcoxon-rank-sum tests. *n* = number of different fields. Boxes indicate first and third quartiles, bands indicate medians, and whiskers indicate ±1.5 interquartile range. Dotted lines denote means. **i**, **j** Increase of intracellular Ca^2+^ by extracellular Ca^2+^ influx was measured by fluorescence intensity of Fluo-8 (Ex/Em = 490/525). To deplete intracellular calcium storage, TECs (isolate DI-102) were treated with 1 µM of Thapsigargin (TH) before addition of extracellular CaCl2 to the medium. Data points indicate means of 3-replicates, and bars denote standard errors. **j** TECs (isolate DI-102) were preincubated with mouse IgG or blocking anti-integrin α3 antibody, followed by addition of TH and CaCl2. Statistical analysis: linear mixed model. **k** Proposed TEC tube-formation model. TEC tube-formation mechanism requires macropinocytosis and lysosomal exocytosis. Source data are provided as a source data file.

However, no prior report has addressed the question as to whether this mechanism of new lumen formation requires vacuole or macropinosome maturation to a LAMP1-positive lysosomal compartment and lysosomal exocytosis. Analysis of the fate of the TMR-dex-positive vesicles in the TECs indicated that they are trafficked to lysosomal-associated membrane protein 1 (LAMP1)-positive organelles^45,46^ based on the colocalization of LAMP1 with TMR-dex after 3 h (Fig. 6a). LAMP1 is a type I transmembrane protein, in which the N-terminal epitopes normally are present in the luminal area of the lysosome. When lysosomes fuse with the plasma membrane during exocytosis, these N-terminal epitopes are exposed on the cell surface^47^. To test for surface exposure of LAMP1-luminal epitopes in the tubes formed by TECs, adherent TECs that had been pre-incubated with TMR-dex were harvested, and the plasma membranes labeled in suspension with Alexa Fluor 647-conjugated wheat germ agglutinin (WGA). This was followed by incubation of the TECs on Matrigel to allow tube formation for 20 h (Fig. 6b). The cells were then fixed and analyzed by immunofluorescence without permeabilization, using a LAMP1-specific monoclonal antibody (H4A3) that recognizes an epitope in the N-terminal luminal domain^47^ (Fig. 6b). The tubes were positive for the H4A3 epitope located in the LAMP1 N-terminus (green), and macropinocytosed TMR-dex accumulated in the TEC tubes as indicated by Alexa Fluor 647-conjugated WGA (magenta) (Fig. 6c). In cross-sectional images of the tubes (Fig. 6d), TMR-dex was found in the hollow area of the tube, which is presumably the lumen, and TMR-dex was surrounded by the H4A3 N-terminal LAMP1 epitope as well as WGA. Collectively, these results suggest that lysosomal exocytosis contributes to lumen formation in TECs.

To determine whether integrin α3β1 is involved in exocytosis during tube formation, the percent colocalization of macropinocytosed TMR-dex and H4A3 antibody was used to quantitate exocytosis after TECs were incubated with a blocking antibody to integrin α3β1. The cell surface presence of the N-terminal LAMP1 epitope for H4A3 in non-permeabilized cells indicates lysosomal exocytosis. Adherent TECs (isolate from ccf2390) preincubated with TMR-dex were harvested, plated on Matrigel, and incubated with or without 0.5 mM EGTA, followed by fixation and labeling with H4A3 antibody without a cell permeabilization step. Inhibition of exocytosis by the calcium-chelating agent EGTA reduced the colocalization, validating the assay system (Fig. 6e). Similar to the EGTA treatment, incubation with integrin α3β1 blocking antibody reduced colocalization of macropinocytosed TMR-dex and H4A3 antibody, indicating that integrin α3β1 promotes exocytosis during tube formation (Fig. 6f).

### Integrin α3β1 enhances calcium influx, promoting macropinocytosis and tube formation

Increases in intracellular free calcium concentration [Ca^2+^]_i_ are known to trigger both macropinocytosis^48,49^ and lysosomal exocytosis^50,51^. Incubation of TECs with culture media containing the calcium-chelating agent EGTA (0.5 mM) resulted in a significant reduction in the number of macropinosomes per cell (Fig. 6g). In addition, EGTA-containing culture media significantly decreased tube formation of TECs on Matrigel (Fig. 6h). This suggests that extracellular calcium is necessary to increase the intracellular free calcium concentration [Ca^2+^]_i_ and promotes macropinocytosis and tube formation.

Integrins can trigger an increase of intracellular free calcium concentration [Ca^2+^]_i_ by enhancing the influx of extracellular calcium. This process is mediated through the interaction with effector proteins such as calreticulin which binds to the KXGFFKR motif in the cytoplasmic domain of integrin α subunits^33,52,53^. To determine whether the integrin α3β1 pathway regulates calcium influx in TECs, thereby promoting macropinocytosis and tube formation, TECs that had been pre-incubated with either P1B5 (anti-integrin α3 subunit) or control mouse IgG were loaded with Fluo-8 dye. This dye, which crosses the cell membrane and remains inside of cells, can be used as an indicator of [Ca^2+^]_i_ as the fluorescence of Fluo-8 is proportionally enhanced upon binding to intracellular free calcium^54^. In these experiments, to separate the increase in [Ca^2+^]_i_ due to calcium release from intracellular stores, the intracellular calcium stores were depleted with Thapsigargin before addition of CaCl_2_ into the assay medium. The fluorescence intensity of Fluo-8 was increased with a higher concentration of CaCl_2_ in the medium (Fig. 6i), suggesting a corresponding influx of extracellular calcium into the cells. The fluorescence intensity of Fluo-8 was decreased in cells pre-treated with P1B5 (Fig. 6j), indicating blockade of integrin α3β1 function led to a significant reduction of calcium influx. To connect the integrin α3β1 enhancement of calcium influx with the integrin α3β1-promotion of macropinocytosis, TECs treated with anti-integrin α3 antibody were subsequently treated with Thapsigargin and we found that Thapsigargin partially reversed the inhibitory effect of anti-integrin α3 antibody on macropinocytosis supporting a mechanistic link between integrin α3β1-enhanced calcium influx and macropinocytosis (Supplementary Fig. 15). Taken together, these data indicate that integrin α3β1 enhances calcium influx in TECs, and thereby promotes macropinocytosis and tube formation. The tube formation mechanism requiring macropinocytosis coupled with lysosomal exocytosis appears to be a major mechanism of tube formation in the TECs.

## Discussion

The current studies show that overexpression of integrin α3β1 acts as a central regulatory mechanism in the complex network of processes that drive tumor-associated vessel formation in GBM. We identify key mechanisms of action of integrin α3β1 in the TECs as enhancement of macropinocytosis and calcium influx, with the calcium influx acting, at least in part, through stimulation of macropinocytosis. This integrin α3β1-stimulated macropinocytosis appears to be an essential step in the accelerated tube formation that supports neovascularization in the TECs. The data further suggest an integrated mechanistic model of vessel formation in TECs. In this model, the integrin α3β1-mediated calcium influx-stimulated macropinocytosis promotes vessel formation by favoring the maturation of macropinosomes to lysosomal vesicles, trafficking of these vesicles in the polarizing TEC, and exocytosis of the lysosomal vesicle at the surface of a nascent lumen.

The overexpression of integrin α3β1 was identified on microarray analysis of gene expression in TECs (relative to NECs) and on analysis of the levels of the protein in TECs. We also found upregulation of the mRNA and protein for the binding partner (CD151) of integrin α3β1 in TECs. On analysis of the expression of the integrin α3 subunit in tissue microarrays, we found significantly higher expression of the integrin α3 subunit on TECs in GBM as compared to ECs in normal brain tissue. Upregulation of integrin α3β1 and CD151 in tumor cells in GBM have been reported to be prognostic markers of poor survival^55^. The concept that this upregulation of integrin α3β1 is a driver of tumor-associated angiogenesis is supported by the observation that blockade of integrin α3β1 greatly reduced sprout formation, implicating α3β1 in this aspect of angiogenesis that likely expands the tube networks, and tube formation. Moreover, blockade of integrin α3β1 reduced vascular density in organotypic cultures of GBM tumor, probably by targeting both vessel formation and angiogenesis. The reduced vessel density observed on blockade of integrin α3β1 could be due to the destabilization of the existing tumor vasculature, potentially hindering the binding of integrin α3β1 with secondary ligands that promote cell-cell interactions.

While the current studies indicate that upregulation of integrin α3β1 in TECs plays a major role in neovascularization in GBM, they do not exclude the possibility that upregulation of other genes in the TECs contributes to angiogenesis either independently of upregulation of α3β1 expression or in concert with it. For example, the upregulated integrin α7 subunit in TECs that pairs with β1, and we show to promote tube formation but not sprout generation in the TECs, will be pursued in future studies. Also, our microarray findings suggest a gene signature indicative of cell-matrix remodeling, and previously others have reported upregulation of SPARC, which regulates cell adhesion^27,56^, and TEM7 (PLXDC1), which regulates cell spreading through an interaction with nidogen^26,57^ in TECs isolated from GBM tumors. Changes in SPARC and TEM7 expression can be part of a gene signature indicative of cell-matrix remodeling, as can TEM8 that promotes cell spreading and is differentially expressed in TECs in multiple non-brain cancers^58–60^. The striking reduction in expression of VE-cadherin in TECs (Fig. 2) also likely contributes to both the greater sprouting and accelerated tube formation. VE-cadherin-mediated intercellular interactions are known to suppress sprouting during embryonic development of zebrafish^61^, and integrin activation negatively regulates VE-cadherin-containing adherens junctions^62^. Although VE-cadherin and integrin α3β1/CD151 are known to transcriptionally regulate the expression of N-cadherin in EC lines^63^ and PTPmu in epithelial cells^64^, respectively, we did not find changes in the expression of either of these genes (Fig. 2b, d). Similarly, although mTORC1 and mTORC2 (mTORC1/2) have been shown to promote angiogenesis^65,66^, our data suggest that integrin α3β1 is not a major driver of mTORC1/2 activity in the experimental conditions of our in vitro studies. Downregulation or functional blockade of integrin α3β1 did not significantly alter the phosphorylation of the mTORC1 substrate S6 kinase, or phosphorylation of the mTORC2 substrate SGK1 (Supplementary Fig. 12a–c).

Our in vitro analyses suggest that a critical mechanism underlying the ability of upregulation of integrin α3β1 to drive tube formation in the TECs is its promotion of macropinocytosis. We found dramatically higher levels of macropinocytosis in TECs and demonstrate that this is associated closely with an integrin α3β1/CD151 pathway based on downregulation studies and blockade of function approaches. This is consistent with the known ability of integrins to promote macropinocytosis^5^ and cytoskeletal-reorganization through the activation of Cdc42 and Rac1^9^.

In terms of acceleration of tube formation in the TECs, the data suggest that the integrin α3β1/CD151 pathway acts primarily by promoting macropinocytosis and we further demonstrate that macropinocytosis is necessary for tube formation in TECs. This is consistent with the reports that non-selective fluid engulfment by macropinocytosis is associated with tube formation in non-brain ECs^5^. Four major mechanisms of tube formation have been identified that can occur concurrently or in a tissue type- or developmental stage-dependent manner:^4^ (i) “cell hollowing” that involves formation of intracellular vacuoles; (ii) “cord hollowing” that utilizes the extracellular space for lumen formation; (iii) transcellular lumen formation and lumen ensheathment; and (iv) lumen expansion due to hemodynamic forces that shape the apical membrane of single ECs or groups of ECs. Prior studies in normal non-brain ECs have suggested that coalescence of cytoplasmic vacuoles initially formed in individual ECs subsequently span adjacent ECs. EC membranes then surround the coalesced vacuoles thereby generating the formation of new lumen^5^. This mechanistic model of vessel formation is supported by studies in the zebrafish^20,22^. However, no prior report has addressed the question as to whether this mechanism of new lumen formation requires vacuole or macropinosome maturation to a LAMP1-positive lysosomal compartment and lysosomal exocytosis. We show here that macropinocytosed TMR-dex is trafficked to a LAMP1-positive compartment in TECs and is exocytosed. Macropinocytosed TMR-dex was detected in the lumen of new tubes formed on Matrigel and was surrounded by the plasma membrane marker, WGA, and by the N-terminal-LAMP1 epitope (mAb H4A3). Collectively, these results indicate that lysosomal exocytosis has a critical role in the tube formation process in TECs and support the concept that “cell hollowing”, which involves intracellular fusion of vacuoles or macropinosomes^5,20^, is a major but likely not the sole mechanism of tube formation in TECs. In contrast, the expression of integrin α3β1 was significantly lower in normal brain ECs and macropinocytosis was barely detectable, suggesting that the major mechanism underlying tube formation in brain NECs may differ from those in TECs. The β1 integrin subunit has been implicated in lumen formation in normal non-brain ECs and the development of apical-basal polarity on 3D matrices, which requires the activation and signaling of multiple proteins, including Cdc42 and Rac1, polarity proteins (Par3 and Par6b), and the PKC isoforms (PKCε and PKCζ)^5,67–69^. The β1 integrin activates not only Cdc42 and Rac1, but also Par3, and its activation of PLCγ1 results in the generation of diacylglycerol and activation of PKCε^67,70^. Supporting our findings, integrins α3β1 and α7β1 have been shown to promote exocytosis through regulation of the cytoskeleton during neuritogenesis^71^.

Calcium ion-binding genes were significantly enriched among the upregulated genes in TECs. We found that functional blockade of integrin α3β1 significantly inhibited calcium influx which is consistent with the ability of integrin signaling to promote calcium ion influx^33^. Our findings that chelation of calcium ions significantly reduced macropinocytosis and tube formation in the TECs is consistent with the well-established positive regulation of both macropinocytosis and lysosomal exocytosis by intracellular free Ca^2+^ concentration^48–51^. Thus, while increased macropinocytosis in TECs may be attributable in part to direct integrin-induced activation of Cdc42 and Rac1 (Supplementary Fig 16)^9,72^, our data support a role for the integrin-stimulated calcium ion influx in the promotion of macropinocytosis and the tube formation process. The cytoskeletal-reorganization necessary for macropinocytosis is also known to require remodeling of the lipid microenvironment through PI3-kinase and phospholipase C, which are parts of calcium-mediated signaling^73,74^. We have not completely ruled out the possibility that this process may partly rely on calcium release from intracellular stores. Taken together our data suggest that integrin α3β1 in TECs can potentially drive tube formation and the development of polarity in our assays through the enhancement of calcium ion influx. The increased calcium ion influx, together with the activation of Cdc42 and Rac1 drives macropinocytosis, and also promotes exocytosis of macropinosomes that have matured into LAMP-1-positive endocytic vesicles thereby promoting lumen and tube formation.

While the current studies do not shed light on the mechanism by which integrin α3β1 stimulates the calcium ion influx, future studies should investigate a role for calreticulin, a Ca^2+^- binding protein as a signal transducer between activated integrin α3β1 and calcium channels. Calreticulin binds to the KXGFFKR motif in the cytoplasmic domain of integrin α subunits, including integrin α3^53^, and integrin-induced transient elevation of extracellular calcium influx is absent in calreticulin-deficient fibroblasts^52^.

The mechanisms by which integrin α3β1 promotes sprout formation in the TECs may reflect its roles in cytoskeletal reorganization and cell migration^9^. Other investigators^28^ have found increased migration of TECs as compared to NECs. Macropinocytosis has been implicated in the control of cell migration through its regulation of integrin-mediated endocytosis and recycling to the cell surface^75^. Integrin α3β1 and CD151 were co-localized with many TMR-dextran-positive endocytic vesicles (Supplementary Fig. 13), potentially connecting increased macropinocytosis to enhanced cell migration in TECs through integrin α3β1 signaling.

In the PDGF-B-driven syngeneic mouse model of GBM^35^, we found no significant difference in the expression of the integrin α3 subunit on TECs in the tumor and ECs in the normal brain. However, there was an increase in CD151 expression on TECs in the mouse tumors consistent with the report that CD151 plays a role in pathologic angiogenesis in the mouse^36^. Taken together with the reported differences in TEM7 (PLXDC1) expression in the tumor-associated vessels of human and mouse tumors in situ^76^ and the recent report of divergent features in mouse and human brains including differences in astrocyte specialization and gene expression^77^, our observation of differences in the expression of both the integrin α3 subunit and CD151 on TECs in human and mouse GBM strongly suggests critical differences in the regulation of tumor-associated new vessel formation in the human and mouse.

Finally, it should be noted that integrin α3β1 has been postulated to be a repressor of angiogenesis based on knockout of integrin α3 in ECs^78^, but the definitive study of mutation of integrin α3 in mice has not been reported. This is of importance, as the expression levels of one integrin subunit may enforce compensatory or alternative signaling pathways. An example of this is the knockout of the integrin β3 subunit or of the β3/β5 subunits in the mouse that has been shown to result in enhanced angiogenesis^79^, whereas mutation of the integrin β3 subunit in the mouse resulted in defective angiogenesis^80^. Compensatory upregulation of VEGFR2 has also been demonstrated in mice that lack the integrin β3 subunit or the integrin β3/β5 subunits, as well as in the mice lacking the integrin α3 subunit in ECs.

In summary, our findings suggest: (i) A coherent model of the mechanisms underlying the aggressive neovascularization in GBM; (ii) The mechanisms driving accelerated development of tube/vessel formation in the TECs differs from the mechanisms underlying tube/vessel formation in normal brain ECs; and (iii) Upregulation of integrin α3β1 plays a central role in neovascularization in GBM, including both sprout and tube formation, and is upregulated on TECs in human GBM biopsies. Collectively, our studies suggest that targeting integrin α3β1 on the vasculature in GBM could be an effective therapeutic approach.

## Methods

Our research complies with all relevant ethical regulations: the Cleveland Clinic Institutional Review Board (IRB) and the University Hospitals Institutional Review Board approved the studies with human tissue; and the Emory University Institutional Animal Care and Use Committee (IACUC) approved the animal study.

### Human tissues

Fresh GBM tumor biopsy samples that were coded and de-identified were obtained from the Cleveland Clinic Brain Tumor Bank (IRB #2559) and the Brain Tumor Bank of the University Hospitals (IRB #4Y02, 10Z07 and 1307) with Informed Consent in accordance with the guidelines and policies of the Cleveland Clinic Institutional Review Board and the University Hospitals Institutional Review Board, respectively (19 samples). Fresh GBM tumor biopsy samples that were coded and de-identified were also obtained from the Cleveland Clinic Brain Tumor Bank (IRB #12-1010: CASE 9312 BBTC Registry) with a waiver of Informed Consent as this is a minimal risk study, in accordance with the guidelines and policies of the Cleveland Clinic Institutional Review Board (four samples). Paraffin sections of GBM biopsies and of normal autopsy brains that were coded and de-identified were obtained from the Pathology Department of the Cleveland Clinic with a waiver of Informed Consent in accordance with the guidelines and policies of the Cleveland Clinic Institutional Review Board (IRB #14-1427). Human glioma tissue microarrays (GL805a, GL805b, and BS17017a) were purchased from US Biomax, Inc.

### Mouse glioma model

The Nestin-*tva*, *lnk4a*-*arf* −/−transgenic mouse model has been described previously^35^. Nine-to-ten week-old Nestin-*tva*, *lnk4a*-*arf* −/−transgenic mice with a C57BL/6 background (two males and five females) were injected intracranially with vector-infected chicken fibroblasts (DF-1) producing RCAS-PDGF-B virus^35^ in accordance with the guidelines and policies regarding animal use at Emory University (IACUC approval # 2003253). The housing conditions for the mice were as follows: 12 h light/12 h darkness; temperature was 72^o^Fahrenheit; and humidity was 40–50%. For intracranial malignant gliomas the Emory University IACUC requires that the mice be euthanized when they demonstrate neurologic signs. Consistent with the IACUC requirements, the mice for this study were euthanized when neurologic signs were demonstrated. All procedures related to the care, handling and usage of animals in this study were reviewed and approved by the IACUC at Emory University (Protocol #2003253). Freshly harvested tumor-bearing brains were fixed in paraformaldehyde (PFA) at 4 ^o^C for 48 h, immersed in 30% buffered-sucrose at 4 ^o^C for 72 h, frozen in OCT and stored at −70 ^o^C.

### Reagents

Reagents used for human cell propagation and culture were purchased as follows: Attachment Factor (4Z0-210) from Cell Systems; collagen type I (354236) and Matrigel (356231: growth factor reduced and phenol red-free) from Corning; human placenta laminin (L6274) from Sigma–Aldrich; EC medium (EGM-MV BulletKit, CC-3125) from Lonza; M199 medium (MDL07) from Caisson; DMEM medium with 1.8 mM CaCl_2_ (11960044) and Ca^2+^-free DMEM (21068028) from Thermo Fisher Scientific; Neurobasal medium (21103-049) from Life Technologies; and Bac-off (Cell Systems, 4Z0-644); amphotericin B (2.5 µg/ml final, Gibco, 15290-026), heat-inactivated fetal bovine serum (FBS-500HI) and penicillin/streptomycin (100 units/ml final, 100 µg/ml final, 725–100) from Cleveland Clinic Cell Services. CD31 MicroBeads (130-0910935) were from Miltenyi Biotec. Growth factors, chelating agents and inhibitors were: Basic fibroblast growth factor (bFGF; GF0003) from EMDMillipore; recombinant human VEGF 165 (VEGF; 4363-10) BioVision; and EGF (PHG0311) from Life technologies; EGTA (E-4378) and EIPA (A3085) from Sigma Aldrich; and thapsigargin (ab120286) from Abcam.

Processing reagents and antibodies used for immunofluorescence analysis were purchased as follows: NP-40 containing buffer (198596, IGEPAL CA-630) from ICN Biomedicals; Blocking solution (Tris-buffered saline with 0.05% Tween 20, from USB; 5% horse serum, 12446C, from SAFC Biosciences; and 5% bovine serum albumin-BSA, BP1600-100, from Fisher Scientific); sodium citrate (S1699) from Dako; Ca^2+^-free Hank’s BSS (#165-1000) from Cleveland Clinic Cell Services-Media, and VectaShield Antifade mounting medium with DAPI nuclear stain (H-1200, from Vector Laboratories). The PKH26 Red Fluorescent Cell Linker Mini-kit for general cell membrane labeling (MINI26-1KT) was purchased from Sigma–Aldrich, 70-kDa TMR-dex (D1818) and wheat germ agglutinin-Alexa-Fluor-647-conjugate (W32466) from Thermo Fisher Scientific, and Fluo-8 dye-loading solution (ab112129) from Abcam.

### qRT-PCR (quantitative reverse transcription polymerase chain reaction)

NECs (422 and 623) and TECs (ccf2390, ccf2405, ccf2445, and ccf2455) were plated on Matrigel-coated dishes in CSC medium with 10% FBS. Once tubes were formed (24–72 h), total RNA was isolated by the Trizol-Chloroform method and cDNA synthesized by SuperScriptIII (Invitrogen). Quantitative PCR was performed with SYBR-Green PCR master mix (Applied Biosystems) on StepOne PLUS qPCR machine (Applied Biosystems). Relative expression was calculated using the comparative Ct method (ddCt) with GAPDH as the endogenous control. Relative expression analysis for each gene included two or three technical replicates per NEC or TEC isolate. Primer sequences: Integrin α3 subunit-ITGA3.F,TCCGAACCAGCATCCCCACCAT; Integrin α3 subunit-ITGA3.R, ATTTCGGCCGGCAGCTCCTC; CD151.F, GTGGTAGCCTAGAGTCCTGGGGAG; CD151.R, AGCCGGGCTGGAAACGACTC; Integrin β1 subunit-ITGB1.F, AAGGTGGTTTCGATGCCATCATGC; Integrin β1 subunit-ITGB1.R, TGTGGAAAACACCAGCAGCCG; Integrin αv subunit-ITGAV.F, TCGCCGTGGATTTCTTCGTGCC; Integrin αv subunit-ITGAV.R, GCTGGGTGGTGTTTGCTTTGGGA; CD9.F, CCCGTTCGGCCCAGGCTAAG; CD9.R, AGCAATCCCGGCAAGCCAGA; FGFR1.F, CCCTCGGGCAGTGACACCAC; FGFR1.R, ACGGGCATACGGTTTGGTTTGGT.

### Isolation and propagation of ECs

TECs were isolated from fresh human GBM (Supplementary Table 1) as described previously^24^ with slight modification. The tissues were dissociated using the Papain Dissociation System (Worthington Biochemical Corporation) and collected using 40-µm diameter cell strainers (352349, Falcon). CD31^+^ cells were sorted using CD31 MicroBeads with LS Columns (130-042-401) and the MACS MultiStand (No 14281) from Miltenyi Biotec. The TECs were propagated on attachment factor in EC medium supplemented with Bac-off, amphotericin B and penicillin/streptomycin. Cells were harvested with trypsin for passaging and sorted for CD31^+^ expression every two passages. They were not used for experiments after passage 10. Six isolates of normal primary human brain microvascular ECs were purchased from Cell Systems (ACBRI 376, ACBRI 422, and ACBRI 623), Abm (T5458), Cell Biologics (H-6023) and Neuromics (HECO2). NECs were propagated on attachment factor in EC medium as described above for the TECs.

For organotypic culture, GBM tumors were cut into small pieces (~4 mm in diameter) and cultured in Matrigel with neurobasal medium supplemented with EGF (1 ng/ml) and bFGF (1 ng/ml). Tumors were fixed with 10% formalin and embedded in paraffin. In all experiments, cells were cultured at 37 °C in a humidified atmosphere with 5% CO_2_.

### Assays of sprout, tube and lumen formation

For tube formation assay of cells in collagen, a collagen gel was prepared using collagen type I (1 mg/ml) and the cells plated on the gel in M199 supplemented +15% FBS, bFGF (15 ng/ml) and VEGF (0.75 ng/ml) for 48 h. A second layer of collagen gel (1 mg/ml) was then plated on top of the cells and after solidification, M199 + 10% FBS was added and the cells cultured for an additional 48 h. For tube formation assay of cells in Matrigel, the Matrigel was solidified in 8-well chamber slides (Falcon culture slides, 354118) according to the manufacturer’s instructions, prior to the addition of cells suspended in M199 + 10% FBS and cultured for the indicated times. Images were acquired using a digital camera (Leica, DFC420) attached to an inverted microscope (Motic, AE21). For live imaging of tube formation, glass bottom microwell dishes (Mattek, P35G-1-14-C) and a Leica DMI6000 inverted microscope were used. Lumen formation was assessed by immunofluorescence staining for laminin-α5 chain as described below and analyzed using the Leica SP8 confocal microscope.

To determine the effects of EIPA on tube formation, cells were first labeled using the PKH26 Red Fluorescent Cell Linker Mini-kit for general cell membrane labeling. The cells were then resuspended in M199 + 10% FBS containing a final concentration of 50 nM EIPA or vehicle (DMSO) as a control, and plated on Matrigel in 48-well plates (CytoOne Cat# cc7682-7548). Images were acquired using the IncuCyte Zoom microscope (Essen Bioscience) and the numbers of tubes per field counted manually. The Statistical analysis is in the figure legend.

### Gene expression microarray and gene set enrichment analyses

TECs and NECs were isolated from Matrigel using Cell Recovery Solution (BD Biosciences, 354253) when similar densities of tubes were formed on Matrigel based on microscopic analysis, and total RNA was extracted using the RNeasy Mini Kit (Qiagen, 74104). Gene expression microarray (Affymetrix, HG-U219) was performed in the Gene Expression and Genotyping Core of Case Western Reserve University (Cleveland, OH). Genes that were >2 fold-upregulated in the TEC-group as compared to the NEC-group were subjected to gene set enrichment and pathway analysis using DAVID Bioinformatics Resources 6.8 (Laboratory of Human Retrovirology and Immunoinformatics, https://david.ncifcrf.gov) and EASE version 2.0 software^30–32^.

### Immunoblotting

NECs and TECs were plated as monolayers on human placenta laminin (coated at 10 µg/ml) in 6-well plates in M199 + 10% FBS for 3 h and 30 min. Whole cell lysates were prepared using NP-40-lysis buffer (50 mM Tris-HCl, pH 8.0, with 150 mM NaCl, and 1.0% NP-40) supplemented with an EDTA-free protease inhibitor cocktail (11 873 580 001) purchased from Roche. Lysates were separated using disulfide-reduced SDS-PAGE, transferred to PVDF membranes (162-0177) from BioRad, and probed with the indicated primary antibodies and peroxidase-linked secondary antibodies. Signals were detected by chemiluminescence using detection reagents (RPN2209) from GE Healthcare and (K-12045-D20) from UBM Advanstar and autoradiography film (E3018, HyBlot CL) from Denville Scientific. The primary antibodies used were: Anti-integrin α6 subunit (T0919) and anti-CD151 (EP6875) from Epitomics Inc., anti-integrin α3 subunit (21992-1-AP) and anti-integrin β1 subunit (12594-1-AP) from Proteintech; anti-integrin αV subunit (4711P) from Cell Signaling; anti-integrin β3 subunit (ab7167, clone BV4) and anti-VE-cadherin (ab33168) from Abcam; anti-glyceraldehyde-3-phosphate dehydrogenase (GAPDH, sc-365062, clone 6C5), and anti-β-tubulin (sc-101527) from Santa Cruz Biotechnology. The peroxidase-conjugated secondary antibodies (NA9340V and NA931V) were purchased from GE Healthcare.

### Immunofluorescence

For immunofluorescence analysis of tube-forming cells, the plated NECs and TECs were fixed with 4% PFA at 22 ^o^C for the time indicated, permeabilized or not as indicated with 5% NP-40 containing buffer at 22 ^o^C for 30 min, and incubated with Blocking solution at 22 °C for the time indicated. The cells were then incubated with primary antibody at 4 ^o^C for 20 h followed by Alexa-Fluor-conjugated secondary antibody at 22 ^o^C for 1 h. The cells were mounted using VectaShield Antifade mounting medium with DAPI nuclear stain. Images were obtained by Leica SP8 confocal microscope, and the 3-D images were constructed using Velocity 6.3 (Perkin Elmer).

For immunofluorescence analysis of frozen section (GBM tumor biopsies and mouse tumor-bearing brain), sections (5-µm thick) were cut, fixed with PFA for 30 min, permeabilized with 0.1% Triton X-100 at 22 °C for 20 min, incubated with Blocking solution and subjected to immunofluorescence analysis using the protocol described above. Images were acquired using a LEICA DM5500B microscope. The images of mouse CD31 were converted to black and white binary images, and images of the mouse integrin α3 subunit, integrin α6 subunit, or CD151 were converted to gray scale images using ImageJ (1.51 s). Converted pairs of images were multiplied to generate mean gray values (average fluorescence intensities) on a CD31-positive pixel.

For immunofluorescence analysis of tissues fixed in paraffin (organotypic cultures and human glioma tissue microarrays), after paraffin removal with xylene washes, sections (5-µm) were subjected to antigen retrieval (10 mM sodium citrate at 95 °C for 20 min) and then incubated with Blocking solution and subjected to immunofluorescence analysis using the protocol described above. For organotypic cultures, images were acquired using the LEICA DM5500B Microscope System, and the percent CD31-positive area per field was obtained using Image J (1.51 s). For human glioma tissue microarrays, images were acquired using a slide scanner (Leica DM6000B) in Dr. George F. Muschler’s laboratory (Cleveland Clinic). The scanned images of vwf were converted to black and white binary images, and the scanned images of the integrin α3 subunit, integrin α6 subunit, or CD151 were converted to gray scale images using ImageJ (1.51 s). Converted pairs of images were multiplied to generate mean gray values (average fluorescence intensities) on a vwf-positive pixel. Only normal brains and GBM tumors in the microarrays were analyzed. The *p* values were obtained by Wilcoxon rank sum test using R programming language, and the graphs were generated by GraphPad Prism 5.

The primary antibodies used for immunofluorescence analysis of human cells and tissues were purchased as follows: Rabbit anti-CD31 antibody (NBP1-71663) from Novus Biologicals; mouse monoclonal anti-integrin α3 subunit (MAB1952z, clone P1B5); rabbit anti-vwf (AB7356) from Millipore; rabbit anti-VE-Cadherin antibody (ab33168), rabbit anti-integrin α3 subunit (ab131055), rabbit anti-CD151 (ab201174), mouse anti-vwf (ab68545), rabbit anti-SNX5 antibody (ab180520, clone EPR14368) and rabbit anti-LAMP1 antibody (ab24170) from Abcam; rabbit anti-integrin α6 subunit (T0919) from Epitomics; goat anti-laminin α5 IgG (sc-16592) from Santa Cruz, and mouse anti-LAMP1 antibody (H4A3) from the Developmental Studies Hybridoma Bank. Secondary antibodies were: Alexa-Fluor-594-conjugated goat anti-mouse IgG (A11032), Alexa-Fluor-594-conjugated donkey anti-goat IgG (A11058), Alexa-Fluor-594-conjugated goat anti-rabbit IgG (A11037) and Alexa-Fluor-488-conjugated goat anti-mouse IgG (A21121) from Thermo Fisher Scientific; and Alexa-Fluor-488-conjugated goat anti-rabbit IgG (A11034) from Life Technologies. For immunofluoresence analysis of mouse glioma, the primary antibodies used were rabbit anti-integrin α3 subunit (Millipore-Sigma, AB1920), rabbit anti-integrin α6 subunit (Bioss, BS-2641R), rabbit anti-CD151 (Epitomics, 5901-1, clone EP6875) and rat anti-mouse CD31 (Dianova, DIA-310) and the secondary antibodies were Alexa-Fluor-568-conjugated goat anti-rabbit IgG (A11036) and Alexa-Fluor-488-conjugated goat anti-rat IgG (A21208) from ThermoFisher Scientific. Manders’ Coefficients were obtained using ImageJ Plugin (JACoP)^81^.

### Treatment with blocking antibodies

NECs and TECs were pre-incubated in suspension with a function blocking antibody (5 µg/ml in experimental medium) on ice for 10–15 min, and then plated on Matrigel in the presence of the blocking antibody (5 µg/ml in experimental medium). The function blocking antibodies used were mouse mAb anti-integrin α3 subunit (EMD Millipore, MAB1952P, clone P1B5), rat anti-integrin α6 subunit (R&D systems, MAB13501, clone GoH3), and mouse mAb anti-CD151 (clone 1A5, a kind gift from Dr. Andries Zijlstra and Dr. James P. Quigley). Normal mouse IgG (SC-2025) and normal rat IgG (sc-2026) from Santa Cruz Biotechnology were used as controls. To analyze the effects of blocking antibodies on organotypic cultures, the organotypic culture were injected on day 2 and day 6 with either mouse IgG or mouse mAb anti-integrin α3 subunit blocking antibody (P1B5). The antibodies were injected at three places to achieve ~5 µg/ml per piece of tumor based on an estimated volume of ~33 mm^3^. On day 9, tumors were fixed with 10% formalin and embedded in paraffin.

### Treatment with siRNA

NECs and TECs were resuspended in M199 + 5% FBS and plated on 6-well plates coated with a thin-layer of Matrigel. On the following day, specific-siRNA or control RNA (Dharmacon, D-001810-02-20, non-targeting siRNA #2) were transfected at 30 nM using Lipofectamine RNAi Max (Thermo Fisher Scientific, 13778075) in Opti-MEM I Reduced Serum Medium (Cleveland Clinic Cell Services-Media, 35-500) for 48 h, and the transfected cells were then lysed for immunoblotting analysis. The siRNAs were custom-made by Sigma–Aldrich: siintegrin α3-1, GCUACAUGAUUCAGCGCAA(dTdT); siintegrin α3-2, GUUUGAAGGCUUGGGCAAA(dTdT); siCD151, CAUGUGGCACCGUUUGCCU(dTdT). To determine the effects of siRNA treatment on macropinocytosis, at 48 h post-transfection, the cells were incubated with 50 µg/ml TMR-dex for 15 min at 37 °C in M199 + 10% FBS.

### Macropinocytosis assays

Uptake of 70-kDa TMR-dex was measured as described previously^39,40^. NECs and TECs adherent to a collagen I (10 µg/ml)-coated surface were incubated with TMR-dex (25 µg/ml) for 30 min in M199 + 5% FBS, followed by washing with PBS and fixation in PFA. To determine the effects of blocking integrin α3β1 or CD151 function on macropinocytosis, cells adherent to collagen I were incubated for 3 h in M199 + 5% FBS in the presence of 5 µg/ml function blocking antibody or control IgG, prior to incubation with TMR-dex in the same blocking antibody-containing medium. TMR-dex particles with diameters ≥0.2 µm were counted using ImageJ. For images with high background, background counts were subtracted based on reference images. Macropinocytosis Index was defined as (total TMR-dex area/total cell area) X 100 per field^40^ and Macropinosome Number was defined as the average number of macropinosomes in a cell per field^41^.

The effects of the macropinocytosis inhibitor, EIPA, on macropinocytosis were determined by preincubating cells adherent to collagen I with EIPA (50 or 100 nM) in M199 + 5% FBS for 10 min and then incubated with TMR-dex in the same medium for 30 min. Images were captured using LEICA DM5500B, and TMR-dex-positive cells or TMR-dex-puncta were counted using ImageJ.

### Lysosome and lysosomal exocytosis assays

To label intracellular LAMP1 and macropinosomes in cells cultured as a monolayer, cells adherent to collagen I were incubated with 25 µg/ml TMR-dex as described above at 37 °C for 3 h. They were then fixed with PFA for 20 min, permeabilized with 0.3% Triton X-100 for 20 min, incubated in Blocking solution, and reacted with anti-LAMP1 antibody, followed by Alexa-Fluor-488-conjugated goat anti-rabbit IgG.

To detect the N-terminal LAMP1-epitope on EC tubes on Matrigel, cells were cultured on collagen I (10 µg/ml)-coated plate in M199 + 5% FBS, and the next day, treated with 100 μg/ml TMR-dex for 15 min at 37 °C in M199 + 5% FBS. Cells were then trypsinized, washed twice with PBS, incubated in suspension with 5 μg/ml wheat germ agglutinin-Alexa-Fluor-647-conjugate at 22 °C for 10 min, washed with PBS, and plated on Matrigel in M199 + 5% FBS in chamber-slides and maintained at 37 ^o^C, 5% CO_2_ for 20 h. Cells were then fixed with PFA for 40 min. The cells were not permeabilized. Slides were incubated in Blocking solution for 2 h, reacted with 2 μg/ml mouse anti-LAMP1 antibody at 4 ^o^C for 20 h, followed by reaction with Alexa-Fluor-488-conjugated goat anti-mouse antibody at 22 ^o^C for 1 h. Images were captured using a Leica SP8 confocal microscope. Velocity 6.3 (PerkinElmer) was used to construct three-dimensional images and cross-sectional images.

### Measurement of calcium influx and effects of calcium ion concentration

The effects of EGTA on tube formation were determined by plating the cells on Matrigel in control medium (DMEM with Ca^2+^ + 5% FBS) for 1 h, after which the culture medium was replaced with 0.5 mM EGTA medium (Ca^2+^-free DMEM + 5% FBS containing 0.5 mM EGTA). Control cells were maintained in control medium. Images were acquired using IncuCyte Zoom (Essen Bioscience) and analyzed with Image J using the Angiogenesis Analyzer Plug-in. The combined numbers of branches and master segments per field in the ImageJ Angiogenesis Plug-in was used to calculate the numbers of tubes per field.

The effects of EGTA on macropinocytosis were determined by plating the cells on collagen I (10 μg/ml)-coated 8-well chamber slides (Falcon culture slides, 354118) in M199 + 5% FBS. The next day the cells were incubated with either control medium or 0.5 mM EGTA medium for 15 min at 37 °C, followed by addition of 100 μg/ml TMR-dex at 37 °C for 15 min and fixation in PFA. Images were acquired using a LEICA DM5500B, and the images analyzed with ImageJ using the Angiogenesis Analyzer Plug-in and GraphPad Prism 5.

To measure calcium influx, after cell harvest with trypsinization and washing with DMEM + 5% FBS, cells were incubated in suspension with either 5 µg/ml blocking antibody or control IgG on ice for 15 min, and then plated on 96-well plates (Falcon, 353072) coated with a thin-layer of Matrigel in DMEM + 5% FBS in the presence of either 5 µg/ml blocking antibody or control IgG. After 2 h of adhesion to the Matrigel at 37 °C and 5% CO_2,_, cells were incubated with 0.5 mM EGTA medium for 10 min at 37 °C, and then incubated with Fluo-8 dye-loading solution for 20 min at 37 °C, followed by washing with Ca^2+^ free Hank’s BSS. Fluo-8 fluorescence was measured using a Cytation5 plate reader and Gen5 software (version 3.00.19) from BioTeck Instruments, Inc., while cells were incubated at 22 °C in the presence of blocking antibody or control IgG in Ca^2+^-free DMEM + 0.01% BSA. Thapsigargin (final concentration, 1 µM) was added at the 3 min-time point after the start of fluorescence reading, and CaCl2 was added at the 11 min-time point. The intensity of fluorescence of Fuo-8 (Ex/Em = 490/525) corresponds to the increase in intracellular calcium.

### Reporting summary

Further information on research design is available in the Nature Research Reporting Summary linked to this article.

## Supplementary information

Supplementary Information

Reporting Summary

## Data Availability

The gene expression microarray data have been deposited in the GEO public database (GSE137902;). Source data underlying Figs. (1a–d, 2a–f, 2h, i, and 3a–d, 4c, 5a–f, and 6e–j) and Supplementary Figures (2b, 3a & b, 4, 6b, 7b, 8b, 10, 12a–c, 14a, 15a, and 16a) are provided as Source Data files in the Supplemental Information. Source data are provided with this paper.

## Source data

Source Data

## References

[1] Gladson CL, Prayson RA, Liu WM (2010). The pathobiology of glioma tumors. Annu Rev. Pathol..

[2] Batchelor TT, Reardon DA, de Groot JF, Wick W, Weller M (2014). Antiangiogenic therapy for glioblastoma: current status and future prospects. Clin. Cancer Res..

[3] Wolburg H, Noell S, Fallier-Becker P, Mack AF, Wolburg-Buchholz K (2012). The disturbed blood-brain barrier in human glioblastoma. Mol. Asp. Med..

[4] Betz C, Lenard A, Belting HG, Affolter M (2016). Cell behaviors and dynamics during angiogenesis. Development.

[5] Davis GE, Stratman AN, Sacharidou A, Koh W (2011). Molecular basis for endothelial lumen formation and tubulogenesis during vasculogenesis and angiogenic sprouting. Int Rev. Cell Mol. Biol..

[6] Lim JP, Gleeson PA (2011). Macropinocytosis: an endocytic pathway for internalising large gulps. Immunol. Cell Biol..

[7] Weis SM, Cheresh DA (2011). Tumor angiogenesis: molecular pathways and therapeutic targets. Nat. Med.

[8] Avraamides CJ, Garmy-Susini B, Varner JA (2008). Integrins in angiogenesis and lymphangiogenesis. Nat. Rev. Cancer.

[9] Huttenlocher A, Horwitz AR (2011). Integrins in cell migration. Cold Spring Harb. Perspect. Biol..

[10] Chandrasekaran L (2000). Cell contact–dependent activation of α3β1 integrin modulates endothelial cell responses to thrombospondin-1. Mol. Bio Cell.

[11] Domínguez-Jiménez C (2001). Involvement of α3 integrin/tetraspanin complexes in the angiogenic response induced by angiotensin II. FASEB J..

[12] Fukushi J, Makagiansar IT, Stallcup WB (2004). NG2 proteoglycan promotes endothelial cell motility and angiogenesis via engagement of galectin-3 and alpha3beta1 integrin. Mol. Biol. Cell.

[13] Nabors LB (2015). Two cilengitide regimens in combination with standard treatment for patients with newly diagnosed glioblastoma and unmethylated MGMT gene promoter: results of the open-label, controlled, randomized phase II CORE study. Neuro Oncol..

[14] Wang N, Jain RK, Batchelor TT (2017). New directions in anti-angiogenic therapy for glioblastoma. Neurotherapeutics.

[15] Wang R (2010). Glioblastoma stem-like cells give rise to tumour endothelium. Nature.

[16] Ricci-Vitiani L (2010). Tumour vascularization via endothelial differentiation of glioblastoma stem-like cells. Nature.

[17] Scully S (2012). Transdifferentiation of glioblastoma stem-like cells into mural cells drives vasculogenic mimicry in glioblastomas. J. Neurosci..

[18] Sigurbjörnsdóttir S, Mathew R, Leptin M (2014). Molecular mechanisms of de novo lumen formation. Nat. Rev. Mol. Cell Biol..

[19] Strilić B (2009). The molecular basis of vascular lumen formation in the developing mouse aorta. Dev. Cell.

[20] Kamei M (2006). Endothelial tubes assemble from intracellular vacuoles in vivo. Nature.

[21] Blum Y (2008). Complex cell rearrangements during intersegmental vessel sprouting and vessel fusion in the zebrafish embryo. Dev. Biol..

[22] Yu JA, Castranova D, Pham VN, Weinstein BM (2015). Single-cell analysis of endothelial morphogenesis in vivo. Development.

[23] Gebala V, Collins R, Geudens I, Phng LK, Gerhardt H (2016). Blood flow drives lumen formation by inverse membrane blebbing during angiogenesis in vivo. Nat. Cell Biol..

[24] Huang P (2012). Endothelial expression of TNF receptor-1 generates a proapoptotic signal inhibited by integrin α6β1 in Glioblastoma. Cancer Res..

[25] Kikkawa Y (2017). Identification of laminin α5 short arm peptides active for endothelial cell attachment and tube formation. J. Pept. Sci..

[26] Beaty RM (2007). PLXDC1 (TEM7) is identified in a genome-wide expression screen of glioblastoma endothelium. J. Neurooncol..

[27] Pen A, Moreno MJ, Martin J, Stanimirovic DB (2007). Molecular markers of extracellular matrix remodeling in glioblastoma vessels: microarray study of laser-captured glioblastoma vessels. Glia.

[28] Charalambous C (2005). Interleukin-8 differentially regulates migration of tumor-associated and normal human brain endothelial cells. Cancer Res..

[29] Madden SL (2004). Vascular gene expression in nonneoplastic and malignant brain. Am. J. Pathol..

[30] Huang DW, Sherman BT, Lempicki RA (2009). Systematic and integrative analysis of large gene lists using DAVID bioinformatics resources. Nat. Protoc..

[31] Huang DW, Sherman BT, Lempicki RA (2009). Bioinformatics enrichment tools: paths toward the comprehensive functional analysis of large gene lists. Nucleic Acids Res..

[32] Hosack DA, Dennis G, Sherman BT, Lane HC, Lempicki RA (2003). Identifying biological themes within lists of genes with EASE. Genome Biol..

[33] Campbell, I. D. & Humphries, M. J. Integrin structure, activation, and interactions. *Cold Spring Harb Perspect Biol* **3** (2011). 10.1101/cshperspect.a004994 PMC3039929 21421922

[34] Yauch RL, Kazarov AR, Desai B, Lee RT, Hemler ME (2000). Direct extracellular contact between integrin α3β1 and TM4SF protein CD151. J. Biol. Chem..

[35] Hambardzumyan D, Amankulor NM, Helmy KY, Becher OJ, Holland EC (2009). Modeling adult gliomas using RCAS/t-va technology. Transl. Oncol..

[36] Takeda Y (2007). Deletion of tetraspanin Cd151 results in decreased pathologic angiogenesis in vivo and in vitro. Blood.

[37] Zhang XP (1999). Alpha 3 beta 1 adhesion to laminin-5 and invasin: critical and differential role of integrin residues clustered at the boundary between alpha 3 N-terminal repeats 2 and 3. Biochemistry.

[38] Hutter-Schmid B, Kniewallner KM, Humpel C (2015). Organotypic brain slice cultures as a model to study angiogenesis of brain vessels. Front Cell Dev. Biol..

[39] Müller-Greven G (2017). Macropinocytosis of bevacizumab by glioblastoma cells in the perivascular niche affects their survival. Clin. Cancer Res..

[40] Commisso C, Flinn RJ, Bar-Sagi D (2014). Determining the macropinocytic index of cells through a quantitative image-based assay. Nat. Protoc..

[41] Wang JT, Teasdale RD, Liebl D (2014). Macropinosome quantitation assay. MethodsX.

[42] Koivusalo M (2010). Amiloride inhibits macropinocytosis by lowering submembranous pH and preventing Rac1 and Cdc42 signaling. J. Cell Biol..

[43] Zijlstra A, Lewis J, DeGryse B, Stuhlmann H, Quigley JP (2008). The inhibition of tumor cell intravasation and subsequent metastasis via regulation of in vivo tumor cell motility by the tetraspanin CD151. Cancer Cell.

[44] Yoshida S, Pacitto R, Yao Y, Inoki K, Swanson JA (2015). Growth factor signaling to mTORC1 by amino acid-laden macropinosomes. J. Cell Biol..

[45] Luzio JP, Hackmann Y, Dieckmann NM, Griffiths GM (2014). The biogenesis of lysosomes and lysosome-related organelles. Cold Spring Harb. Perspect. Biol..

[46] Caviglia S, Brankatschk M, Fischer EJ, Eaton S, Luschnig S (2016). Staccato/Unc-13-4 controls secretory lysosome-mediated lumen fusion during epithelial tube anastomosis. Nat. Cell Biol..

[47] Andrews NW (2017). Detection of lysosomal exocytosis by surface exposure of lamp1 luminal epitopes. Methods Mol. Biol..

[48] Kabayama H (2009). Ca2+ induces macropinocytosis via F-actin depolymerization during growth cone collapse. Mol. Cell Neurosci..

[49] Canton J (2016). Calcium-sensing receptors signal constitutive macropinocytosis and facilitate the uptake of NOD2 ligands in macrophages. Nat. Commun..

[50] Rodríguez A, Webster P, Ortego J, Andrews NW (1997). Lysosomes behave as Ca2+-regulated exocytic vesicles in fibroblasts and epithelial cells. J. Cell Biol..

[51] Reddy A, Caler EV, Andrews NW (2001). Plasma membrane repair is mediated by Ca(2+)-regulated exocytosis of lysosomes. Cell.

[52] Coppolino MG (1997). Calreticulin is essential for integrin-mediated calcium signalling and cell adhesion. Nature.

[53] Rojiani MV, Finlay BB, Gray V, Dedhar S (1991). In vitro interaction of a polypeptide homologous to human Ro/SS-A antigen (calreticulin) with a highly conserved amino acid sequence in the cytoplasmic domain of integrin alpha subunits. Biochemistry.

[54] McMahon SM, Jackson MB (2014). In situ Ca2+ titration in the fluorometric study of intracellular Ca2+ binding. Cell Calcium.

[55] Zhou P (2015). CD151-α3β1 integrin complexes are prognostic markers of glioblastoma and cooperate with EGFR to drive tumor cell motility and invasion. Oncotarget.

[56] Murphy-Ullrich JE, Sage EH (2014). Revisiting the matricellular concept. Matrix Biol..

[57] Lee HK, Seo IA, Park HK, Park HT (2006). Identification of the basement membrane protein nidogen as a candidate ligand for tumor endothelial marker 7 in vitro and in vivo. FEBS Lett..

[58] Chaudhary A (2012). TEM8/ANTXR1 blockade inhibits pathological angiogenesis and potentiates tumoricidal responses against multiple cancer types. Cancer Cell.

[59] Werner E, Kowalczyk AP, Faundez V (2006). Anthrax toxin receptor 1/tumor endothelium marker 8 mediates cell spreading by coupling extracellular ligands to the actin cytoskeleton. J. Biol. Chem..

[60] van Beijnum JR, Petersen K, Griffioen AW (2009). Tumor endothelium is characterized by a matrix remodeling signature. Front. Biosci. (Sch. Ed.).

[61] Abraham S (2009). VE-Cadherin-mediated cell-cell interaction suppresses sprouting via signaling to MLC2 phosphorylation. Curr. Biol..

[62] Wang Y (2006). Integrins regulate VE-cadherin and catenins: dependence of this regulation on Src, but not on Ras. Proc. Natl Acad. Sci. USA.

[63] Giampietro C (2012). Overlapping and divergent signaling pathways of N-cadherin and VE-cadherin in endothelial cells. Blood.

[64] Chattopadhyay N, Wang Z, Ashman LK, Brady-Kalnay SM, Kreidberg JA (2003). α3β1 integrin–CD151, a component of the cadherin–catenin complex, regulates PTPμ expression and cell–cell adhesion. J. Cell Biol..

[65] Farhan MA, Carmine-Simmen K, Lewis JD, Moore RB, Murray AG (2015). Endothelial cell mTOR Complex-2 regulates sprouting angiogenesis. PLoS One.

[66] Dodd KM, Yang J, Shen MH, Sampson JR, Tee AR (2015). mTORC1 drives HIF-1α and VEGF-A signalling via multiple mechanisms involving 4E-BP1, S6K1 and STAT3. Oncogene.

[67] Zovein AC (2010). Beta1 integrin establishes endothelial cell polarity and arteriolar lumen formation via a Par3-dependent mechanism. Dev. Cell.

[68] Koh W, Mahan RD, Davis GE (2008). Cdc42- and Rac1-mediated endothelial lumen formation requires Pak2, Pak4 and Par3, and PKC-dependent signaling. J. Cell Sci..

[69] Koh W (2009). Formation of endothelial lumens requires a coordinated PKCϵ-, Src-, Pak-and Raf-kinase-dependent signaling cascade downstream of Cdc42 activation. J. Cell Sci..

[70] Jones NP, Peak J, Brader S, Eccles SA, Katan M (2005). PLCγ1 is essential for early events in integrin signalling required for cell motility. J. Cell Sci..

[71] Gupton SL, Gertler FB (2010). Integrin signaling switches the cytoskeletal and exocytic machinery that drives neuritogenesis. Developmental Cell.

[72] Zhang Y (2021). Macropinocytosis in Cancer-Associated Fibroblasts is Dependent on CaMKK2/ARHGEF2 signaling and functions to support tumor and stromal cell fitness. Cancer Disc.

[73] King JS, Kay RR (2019). The origins and evolution of macropinocytosis. Philos. Trans. R. Soc. Lond. B Biol. Sci..

[74] Ghigo A, Laffargue M, Li M, Hirsch E (2017). PI3K and calcium signaling in cardiovascular disease. Circ. Res..

[75] Gu Z, Noss EH, Hsu VW, Brenner MB (2011). Integrins traffic rapidly via circular dorsal ruffles and macropinocytosis during stimulated cell migration. J. Cell Biol..

[76] Carson-Walter EB (2001). Cell surface tumor endothelial markers are conserved in mice and humans. Cancer Res..

[77] Hodge RD (2019). Conserved cell types with divergent features in human versus mouse cortex. Nature.

[78] da Silva RG (2010). Endothelial alpha3beta1-integrin represses pathological angiogenesis and sustains endothelial-VEGF. Am. J. Pathol..

[79] Reynolds LE (2002). Enhanced pathological angiogenesis in mice lacking beta3 integrin or beta3 and beta5 integrins. Nat. Med..

[80] Mahabeleshwar GH, Feng W, Phillips DR, Byzova TV (2006). Integrin signaling is critical for pathological angiogenesis. J. Exp. Med..

[81] Bolte S, Cordelieres FP (2006). A guided tour into subcellular colocalization analysis in light microscopy. J. Microsc..

