# Supplementary material for: Integrin α3β1 promotes vessel formation of glioblastoma-associated endothelial cells through calcium-mediated macropinocytosis and lysosomal exocytosis
Source: Nat Commun. 2022 Jul 25;13:4268. doi: 10.1038/s41467-022-31981-2 (PMC9314429; doi:10.1038/s41467-022-31981-2)
Supplement: Supplementary file 1 — Supplementary Information [file 41467_2022_31981_MOESM1_ESM.pdf]

Supplementary information

Integrin α3β1 promotes vessel formation of glioblastoma-associated endothelial cells through calcium-mediated macropinocytosis and lysosomal exocytosis

Bae et al. 2022

Supplementary Figures

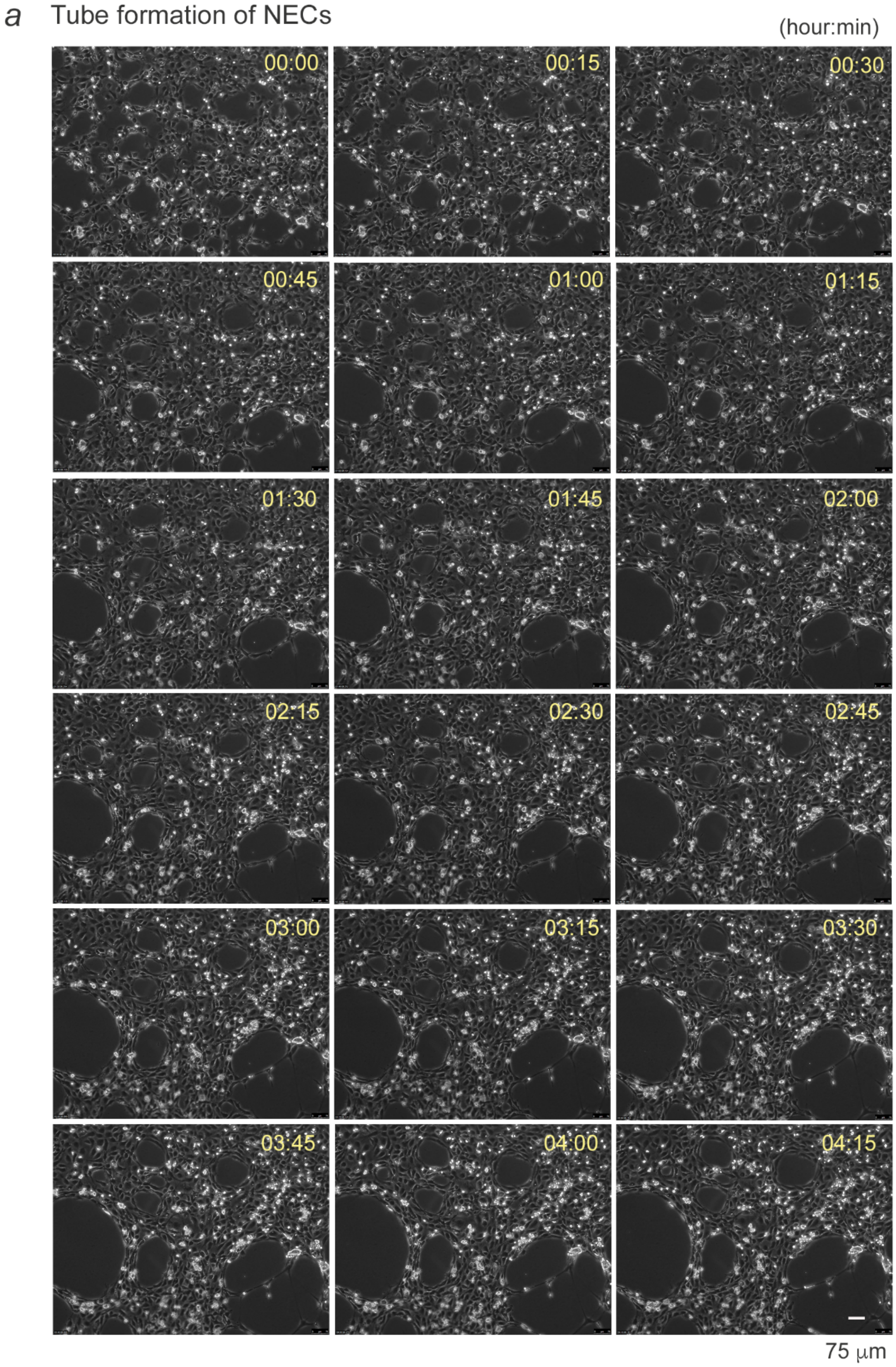

b Tube formation of TECs

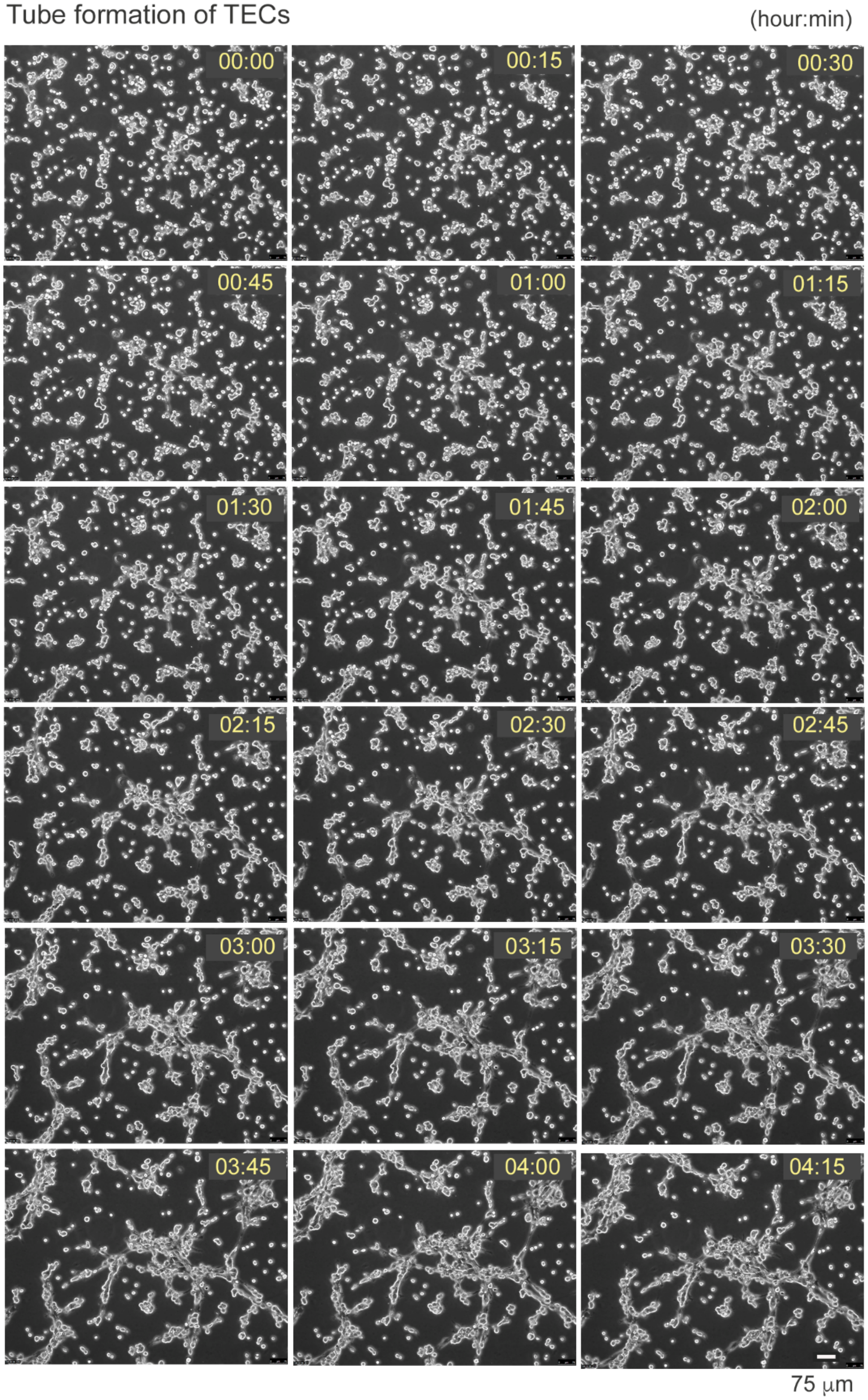

SFig. 1. Live video microscopy of tube formation by TECs and NECs over the first 4 hrs 15min. NEC (376) **(*a*)** and TEC (ccf2515) **(*b*)** were cultured on Matrigel and subjected to live video microscopy over the first 4 hrs 15min of culture.

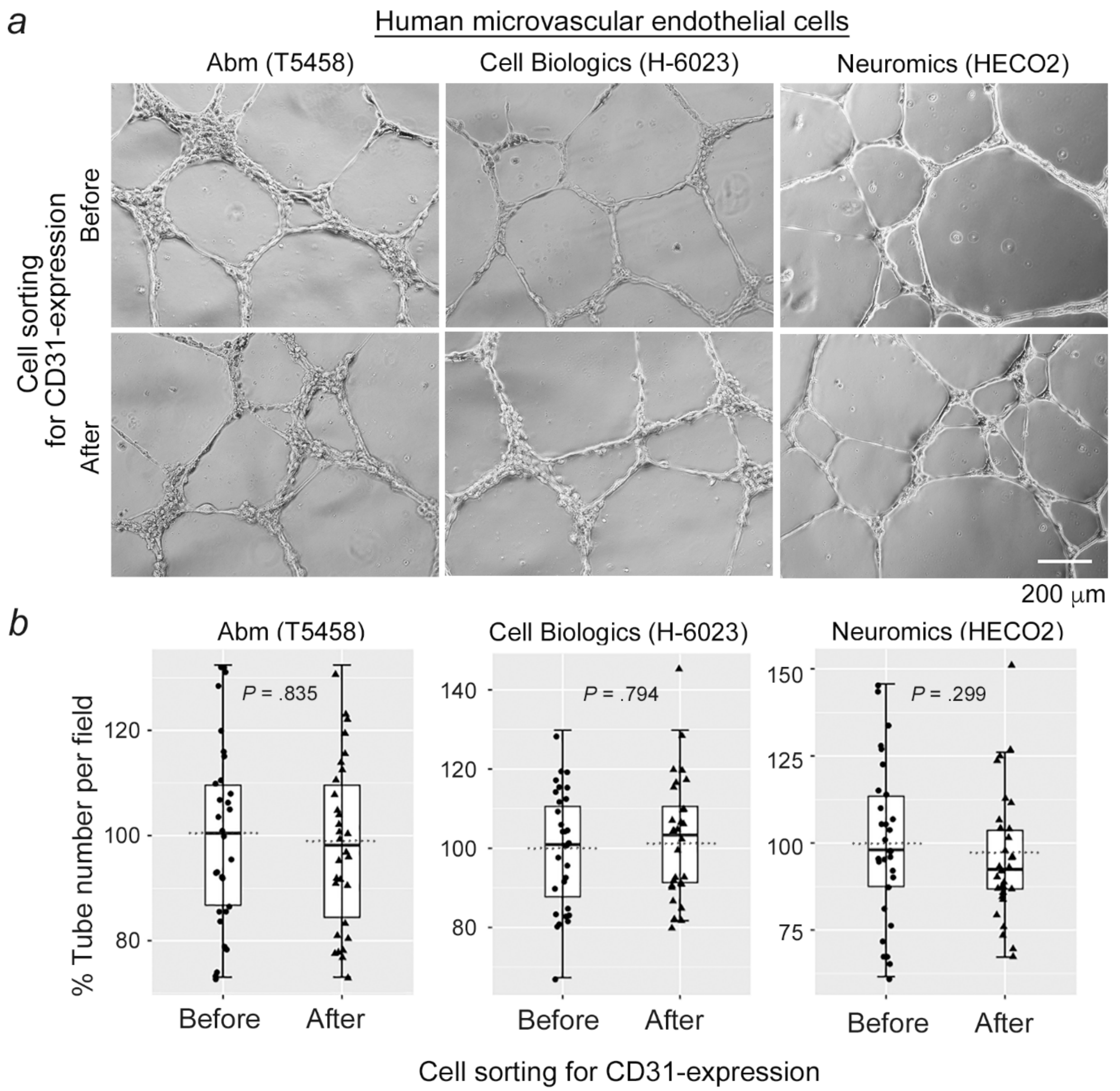

SFig. 2. No effect of cell sorting for CD31 on tube or sprout formation. Tube formation assay on Matrigel was quantitated at 24 h in three additional normal human brain microvascular endothelial cells that were purchased from Abm (T5458), Cell Biologics (H-6023) and Neuromics (HECO2). **(*a*)** The tube formation assay was performed before or after two rounds of cell sorting for CD31-expression using the CD31 MicroBead kit (Miltenyl Biotec Inc., 130-091-935). **(*b*)** Quantitation of the tube formation assay is shown as % tube number per field (100 x the tube number per field/the average number of tubes per field in cells prior to sorting). n denotes the number of different fields. n= 30 in the conditions of before sorting for CD31 and after sorting for CD31 in all three NECs. Boxes indicate first and third quartiles, bands indicate medians, and whiskers indicate ±1.5 interquartile range.

Dotted lines indicate means. Statistical analysis: two sided-Wilcoxon rank-sum test. Source data are provided as a source data file.

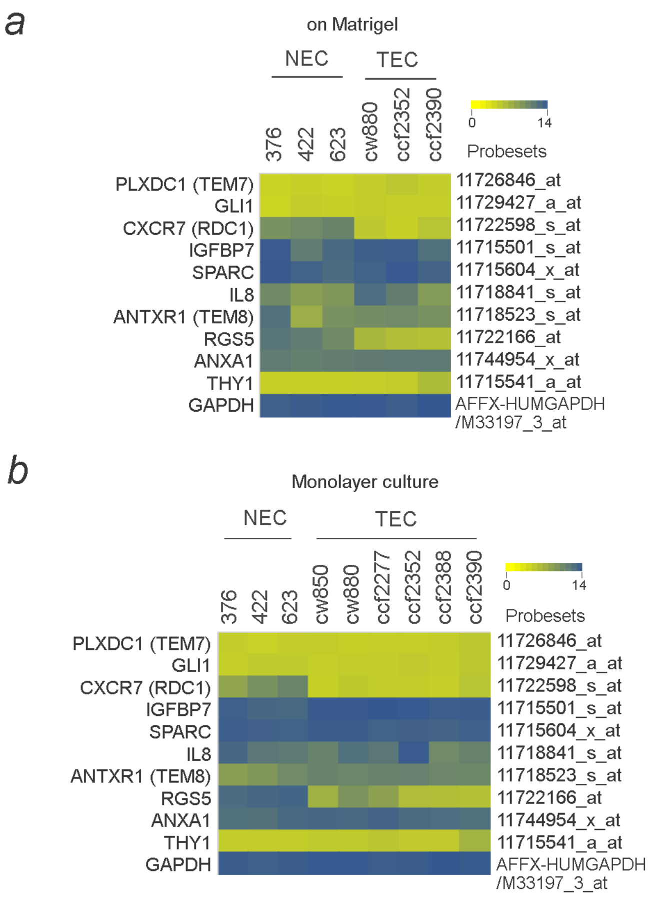

SFig. 3. Expression of TEC markers. Total RNAs were extracted from 3 NECs (376, 422, and 623) and 3 TECs (cw880, ccf2352, and ccf2390) when similar densities of tubes were formed on Matrigel ***(a)*** or from monolayers of 3 NECs (375, 422, and 623) and 6 TECs (cw850, cw880, ccf2277, ccf2352, ccf2388, and ccf2390) (***b***). Gene expression microarrays (Affymetrix Human Genome U219 Array, HG-U219) were performed as described in the Methods. The expression profiles of TEC markers are shown as heat maps. PLXDC1 (plexin domain containing 1), TEM7 (tumor endothelial marker 7), GLI1 (glioma-associated oncogene homolog 1), CXCR7 (C-X-C chemokine receptor type 7), RDC1 (G-protein coupled receptor RDC1 homolog), IGFBP7 (insulin-like growth factor-binding protein 7), SPARC (Secreted protein acidic and rich in cysteine), IL8 (interleukin 8), ANTXR1 (anthrax toxin receptor 1), TEM8 (tumor endothelial marker 8), RGS5 (regulator of G protein signaling 5), ANXA1 (annexin A1), THY1 (thymus cell antigen 1). Source data are provided as a source data file.

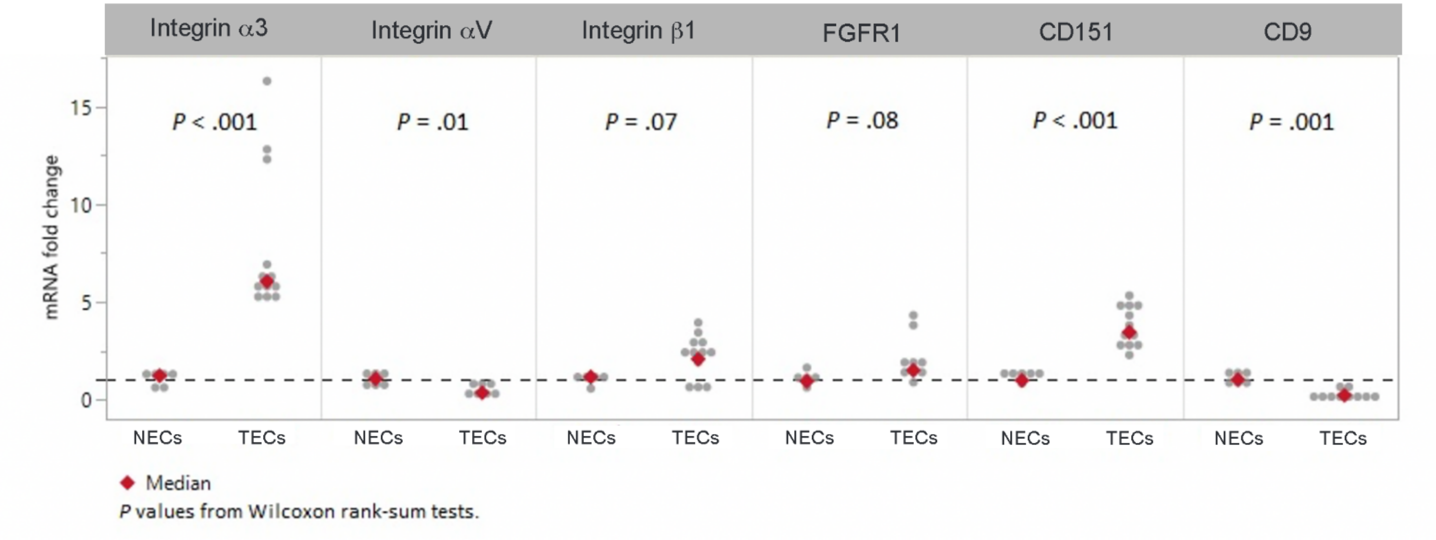

SFig. 4. The mRNA levels of the integrin α3 subunit and CD151 are increased in TECs as compared to NECs. NECs (422 and 623) and TECs (ccf2390, ccf2405, ccf2445, and ccf2455) were plated on Matrigel-coated dishes in CSC medium with 10% FBS. Once tubes were formed (24-72 hrs), total RNA was isolated, and qRT-PCR was performed as described in the methods. n = the number of PCR reactions: For Integrin α3, n=6 for NECs, n=12 for TECs; for Integrin αV, n=6 for NECs, n=7 for TECs; for Integrin β1, n=5 for NECs, n=12 for TECs; for FGFR1 (fibroblast growth factor receptor 1), n=4 for NECs, n=9 for TECs; for CD151 (cluster of differentiation 151), n=6 for NECs, n=11 for TECs; for CD9 (cluster of differentiation 9), n=6 for NECs, n=10 for TECs. Statistical analysis: two-sided Wilcoxon rank-sum test. The exact *P* value for comparisons between NECs and TECs for both integrin α3 and CD151 is *P* = .0001. Source data are provided as a source data file.

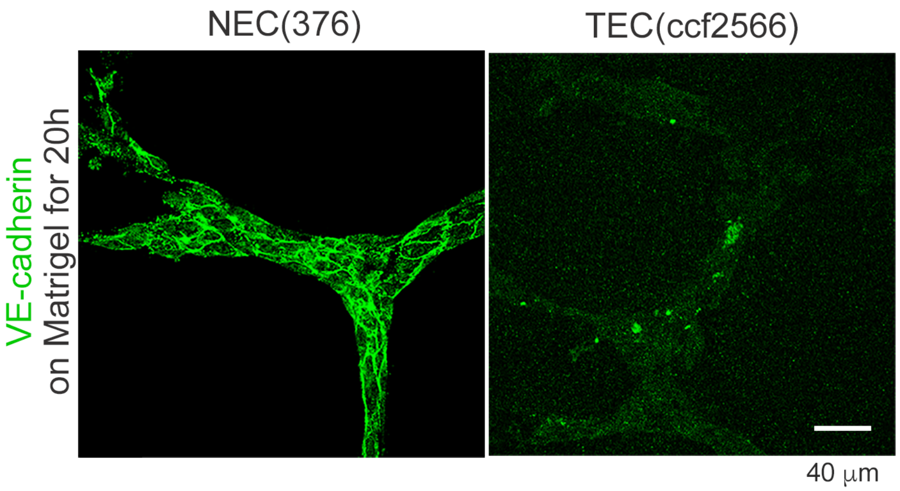

SFig. 5. Decreased expression of VE-cadherin in TECs. TEC (ccf2566) and NEC (376) were cultured on Matrigel in M199 media with 10% FBS for 20 h, and then labeled with anti-VE-cadherin antibody (Abcam, ab33168), followed by Alexa Fluor 488 goat anti-rabbit IgG (Life Technologies, A11034), and DAPI nuclear stain. Cells were imaged on the Leica SP8 confocal microscope. Consistent with the gene expression microarray (Fig. 2b) and the western blotting data (Fig. 2e), the TEC showed markedly decreased VE-cadherin expression overall and VE-cadherin expression appeared largely absent from intercellular junctions. Independent experiments were performed at least two-times with similar results.

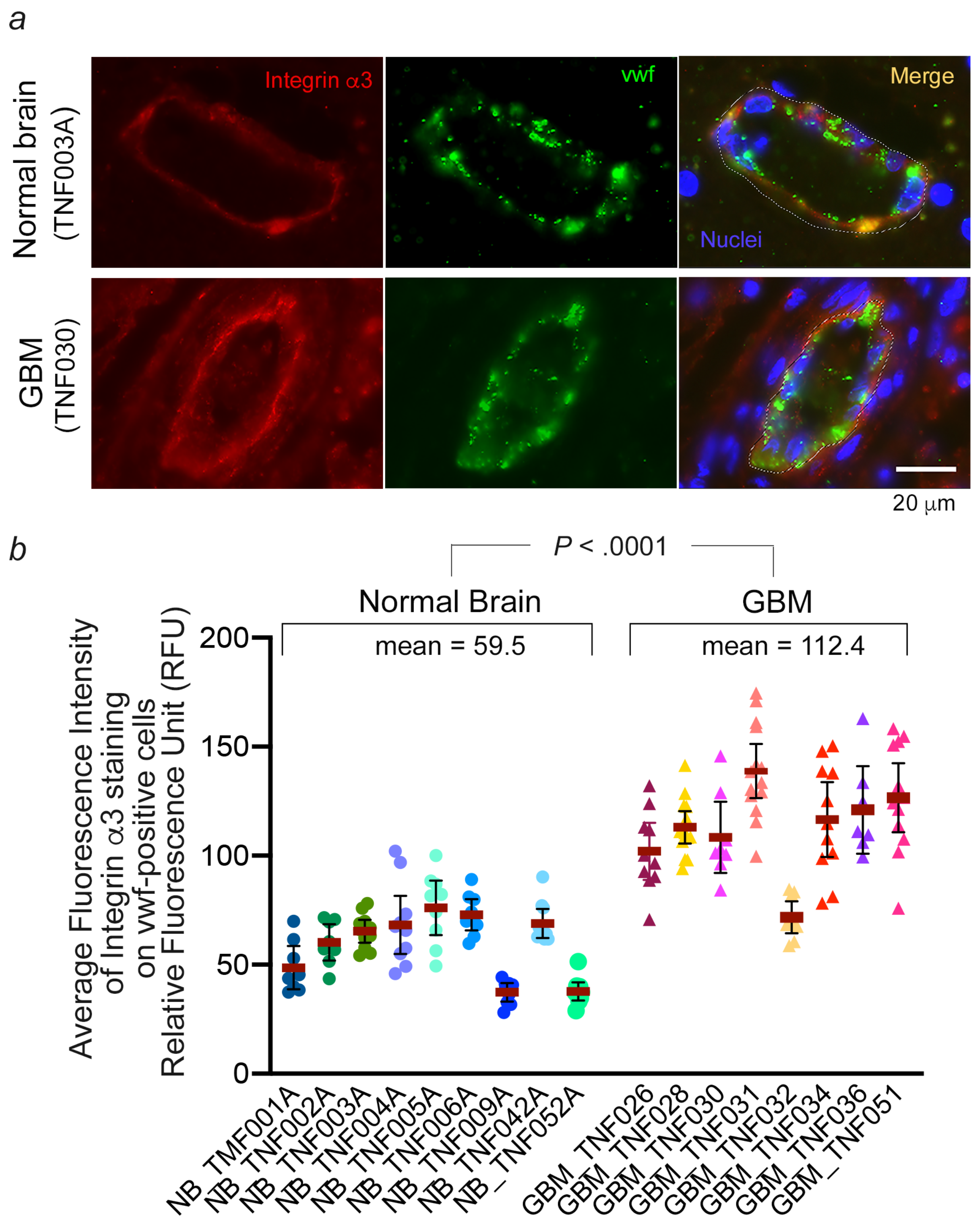

SFig. 6. Increased expression of the integrin α3 subunit in vwf-positive cells in GBM sections. (*a & b*)

Double-label immunofluorescence on paraffin sections from nine normal autopsy brains and eight

GBM biopsies samples was performed with rabbit anti-integrin $\alpha 3$ antibody (Abcam ab131055) and mouse anti-vwf antibody (F8/86, ThermoFisher Scientific, MA5-14029), followed by Alexa Fluor 594-conjugated goat anti-rabbit IgG (Life Technologies, A11037) and Alexa Fluor 488-conjugated goat anti-mouse IgG (Life Technologies, A32723), and DAPI nuclear stain. **(a)** Dotted lines on the merged images indicate vwf-positive cells. The mean fluorescence intensity of integrin $\alpha 3$ staining in vwf-positive cells was quantitated using ImageJ. **(b)** Statistical analysis: a linear mixed model; the data is plotted as a dot plot which shows the medians (red lines) and the 95% confidence intervals (black lines). n = number of different fields: n = 83 for normal brain, and n = 85 for GBM. Paraffin sections of GBM biopsies and of normal autopsy brains were obtained from the Pathology Department at the Cleveland Clinic in a coded and de-identifiable manner, in accordance with the guidelines and policies of the Cleveland Clinic Institutional Review Board. Source data are provided as a source data file.

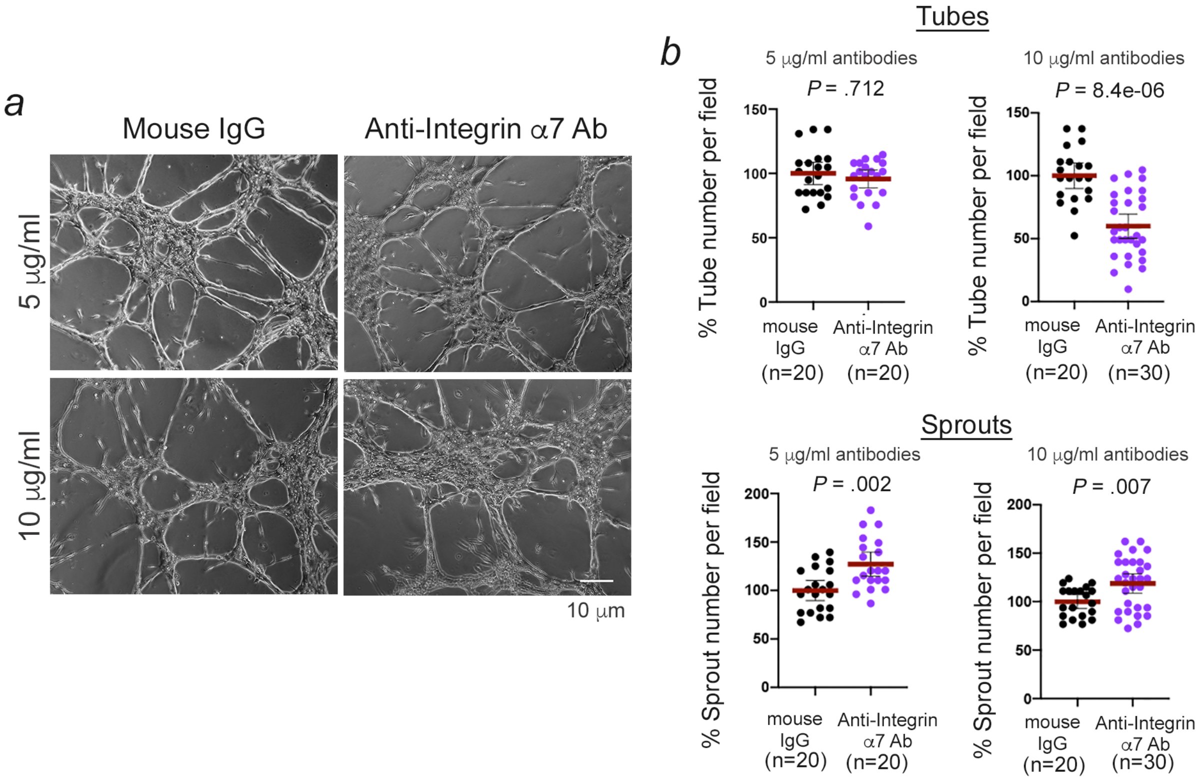

SFig. 7. The effect of a function blocking antibody towards the integrin α7 subunit on the formation of tubes and sprouts by TECs. (a) TECs (isolate from DI-337) were incubated with function blocking antibody towards the integrin α7 subunit (9.1 ITGA7, Developmental Studies Hybridoma Bank-DSHB) or control IgG (mouse IgG, SC-2025, Santa Cruz Biotechnology, Inc.) at 5 or 10 μg/ml on growth factor-reduced Matrigel in M199 medium + 10% FBS for 20 h. The anti-integrin α7 antibody (9.1 ITGA7) was purified from mouse serum using Protein G HP Spin Trap (28-9031-34, GE Healthcare) and Ab buffer kit (28-9030-59, GE Healthcare) according to the manufacturer's instruction. Images were captured by an inverted microscope (Leica DMI6000 SD). Representative images are shown. The bar in the bottom right image represents 10 μm. **(b)** The mean numbers of tubes or sprouts in the mouse IgG-treated group are represented as 100%. Dot plots show % mean of the numbers of tubes or sprouts (red lines) and the 95% confidence interval (black lines). On the x-axis, n = number of different fields. Statistical analysis: two-sided Wilcoxon rank sum tests. Source data are provided as a source data file.

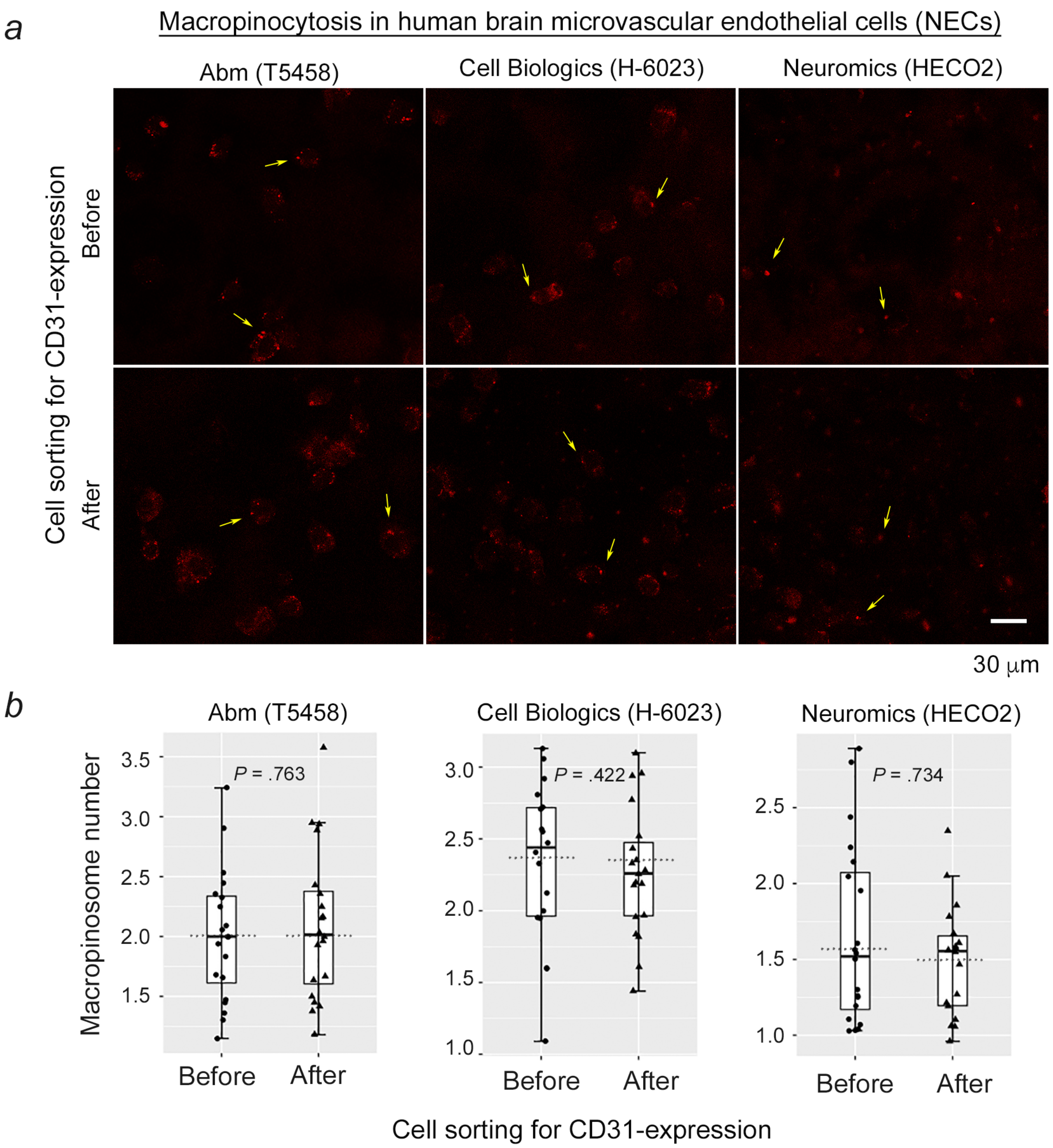

SFig. 8. No effect of cell sorting for CD31 on macropinocytosis in three additional isolates of normal human brain NECs. The three normal human brain microvascular endothelial cells (NECs) (purchased from Abm (T5458), Cell Biologics (H-6023) and Neuromics (HECO2)) were plated on collagen I in M199+5% FBS and incubated with 25 μg/ml TMR-dex for 30 min, and then imaged on the confocal microscope and quantitated for TMR-dex-positive macropinosomes. **(*a*)** The macropinocytosis assay was performed before or after two rounds of cell sorting for CD31-expression using the CD31 MicroBead kit (Miltenyl Biotec Inc., 130-091-935). **(*b*)** Macropinocytosis was quantified as the macropinosome number (the average number of macropinosmes in a cell per field). Dotted lines

indicate means. Boxes indicate first and third quartiles, bands indicate medians, and whiskers indicate ±1.5 interquartile range. n = number of different fields: n = 20 before sorting for CD31 and n=20 after sorting for CD31 in NECs from Abm(T5459); n = 19 before sorting for CD31 and n=19 after sorting for CD31 in NECs from Cell Biologics (H-6023); n = 20 before sorting for CD31 and n = 18 after sorting for CD31 in NECs from Neuromics (HECO2). Statistical analysis: two-sided Wilcoxon rank sum tests. Source data are provided as a source data file.

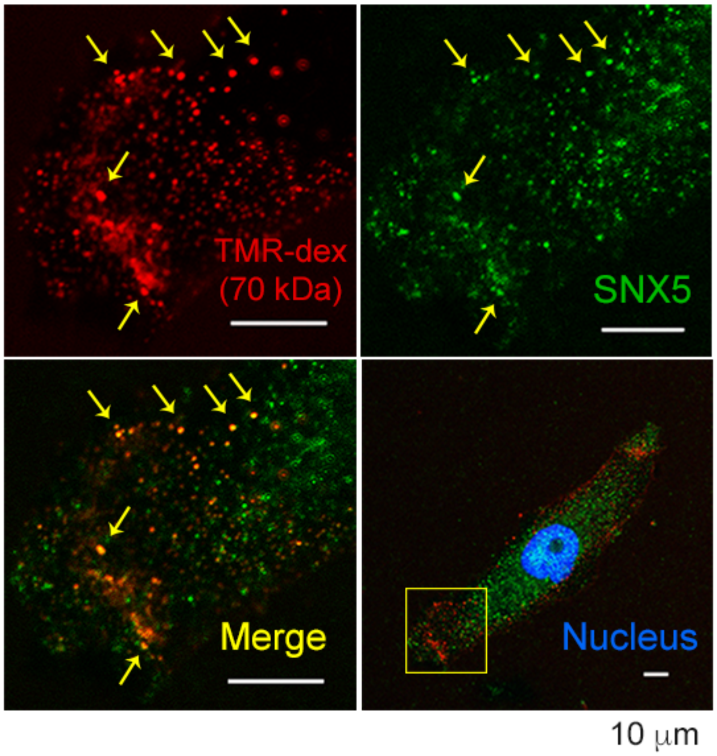

SFig. 9. Many vesicles containing TMR-dex (70kDa) are positive for a macropinosome marker, SNX5. Macropinosomes were visualized by incubating TECs (isolate from ccf2390) briefly with 25 μg/ml TMR-dex for 5 min. The cells were then fixed with PFA and permeabilized with 0.3% Triton X-100. After incubating with Blocking solution, the cells were subject to immunofluorescence analysis using rabbit anti-SNX5 antibody followed by Alexa Fluor 488-conjugated goat anti-rabbit IgG and nuclear staining. Images were acquired using a Leica SP8 confocal microscope. Yellow arrows show colocalization of TMR dextran and SNX5. Independent experiments were performed at least two times with similar results.

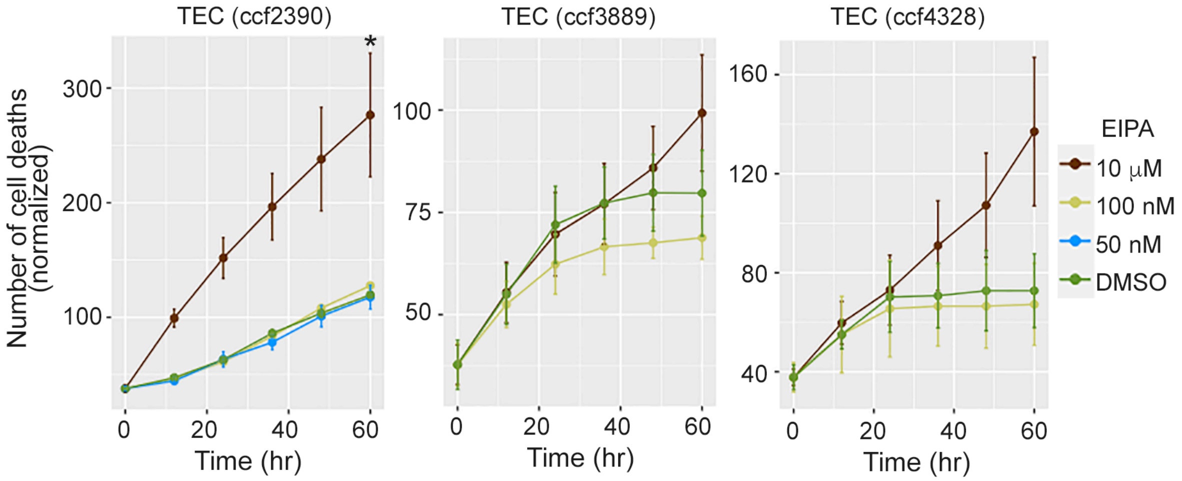

SFIg. 10. No effect of EIPA on cytotoxicity in TECs at the concentration used. TECs (isolates from ccf2390, ccf3889, and ccf4328) were incubated on collagen type I in M199+5%FBS with the cytotoxicity indicator (Promega, G8731) and EIPA at different concentrations as shown or DMSO as vehicle control. Cytotoxicity was assessed using IncuCyte (Essen Bioscience) at the time points indicated, and the numbers of cell death were normalized. n = number of different fields: n = 4 for each data point. Means and standard errors are shown. Statistical analysis: two-sided Wilcoxon rank sum test between DMSO and 10 μM EIPA groups at 60 hr; for ccf2390 $P = .001$ (*), for ccf3889 $P = .49$, and for ccf4328 $P = .11$. At the concentration of EIPA used to inhibit macropinocytosis (50 or 100nM), no increase in cytotoxicity was detected. Source data are provided as a source data file.

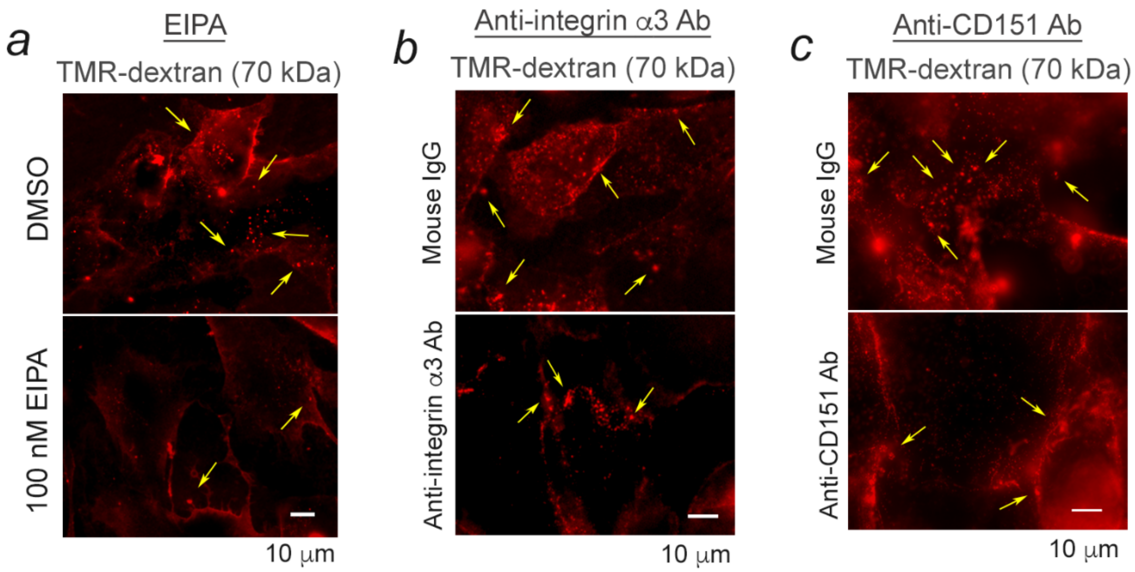

SFig. 11. Inhibition of macropinocytosis by EIPA, mAb anti-integrin α3, or mAb anti-CD151 antibody. (*a*) TECs (isolate from ccf2390) treated with DMSO (vehicle control) or 100 nM EIPA for 10 min were subsequently incubated with TMR-dex for 30 min, followed by imaging. **(*b&c*)** TECs (isolate from ccf2390) were pretreated for 3 h with blocking antibody toward the integrin α3 subunit or mouse IgG **(*b*)**, and TECs (isolate from DI-102) were pretreated with blocking antibody toward CD151 or mouse IgG **(*c*)**, and then TECs were incubated for 30 min with TMR-dex, followed by fixation and imaging. For the macropinocytosis experiments (*a-c*), the culture medium was M199 +5% FBS. Independent experiments were performed at least two times with similar results.

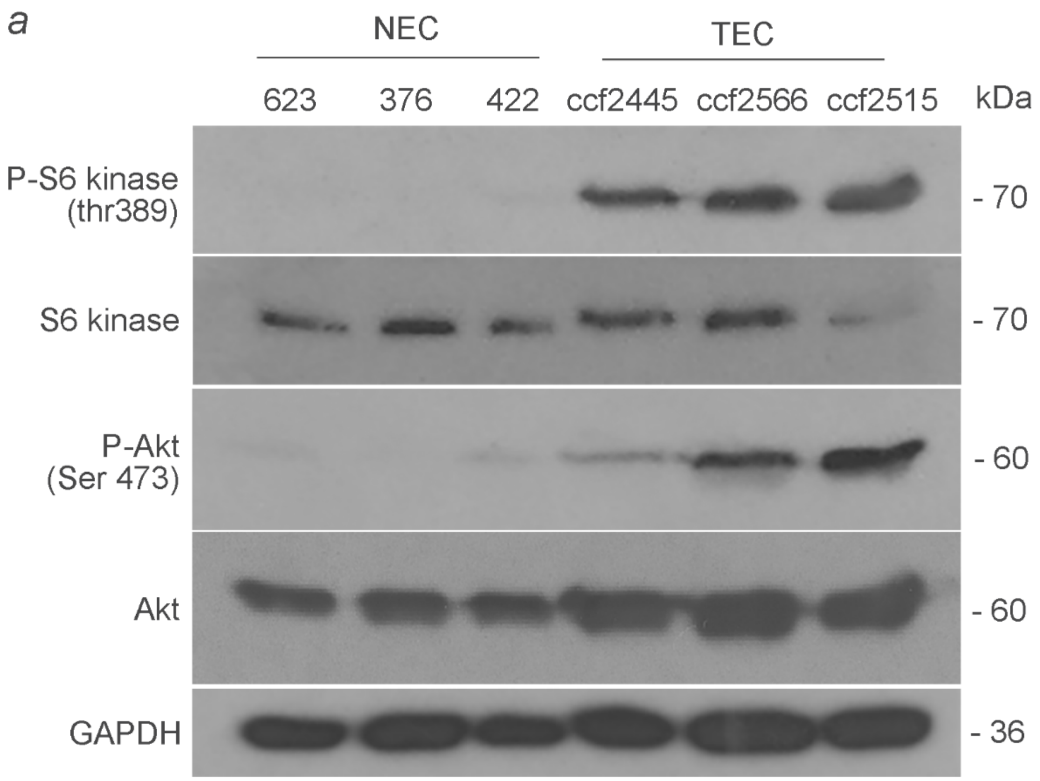
a
NEC
TEC
623
376
422
ccf2445
ccf2566
ccf2515
kDa
P-S6 kinase (thr389)
- 70
S6 kinase
- 70
P-Akt (Ser 473)
- 60
Akt
- 60
GAPDH
- 36

b

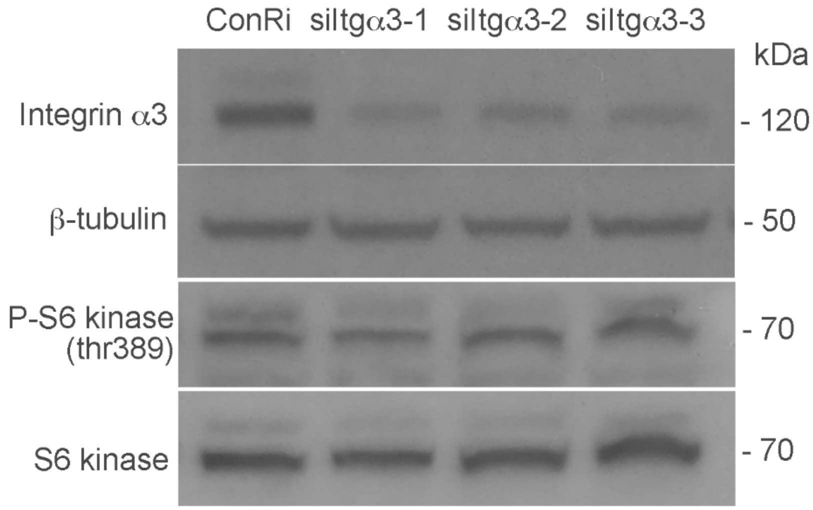
ConRi
siItgα3-1
siItgα3-2
siItgα3-3
kDa
Integrin α3
- 120
β-tubulin
- 50
P-S6 kinase (thr389)
- 70
S6 kinase
- 70

c

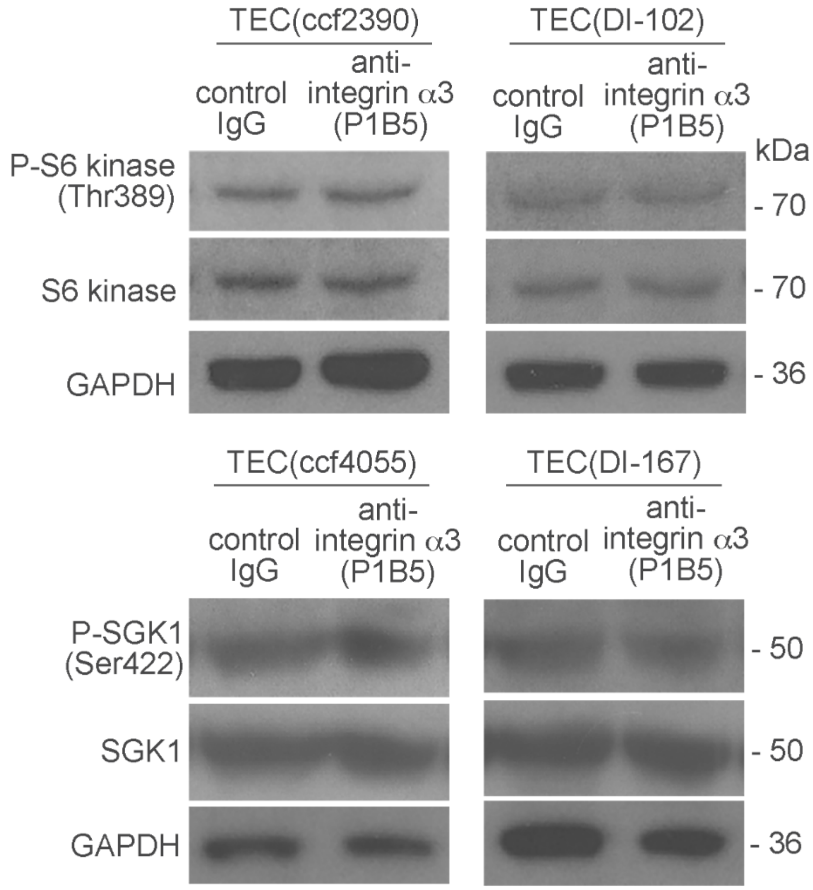
TEC(ccf2390)
TEC(DI-102)
control IgG
anti-integrin α3 (P1B5)
kDa
P-S6 kinase (Thr389)
- 70
S6 kinase
- 70
GAPDH
- 36
TEC(ccf4055)
TEC(DI-167)
control IgG
anti-integrin α3 (P1B5)
P-SGK1 (Ser422)
- 50
SGK1
- 50
GAPDH
- 36

SFig. 12. Elevated mTORC1 signal in TECs is not reduced by siRNA or blocking antibody towards integrin α3β1. (*a*) After adherence to placenta laminin for 3 hours in M199+10%FBS, NECs (623, 376, and 422) and TECs (ccf2445, ccf2566, and ccf2515) were lysed with NP40 lysis buffer. **(*b*)** TEC (ccf2390) were transfected with 30 nM siRNAs or control RNA as described in the Methods. At 48 hours post transfection, cells were lysed for SDS-PAGE and immunoblotting. siRNA sequences: siintegrin α3-1, GCUACAUGAUUCAGCGCAA(dTdT); siintegrin α3-2, GUUUGAAGGCUUGGGCAAA(dTdT); siintegrin α3-3, CCCGATTCCTGGTAGTGAA (dTdT). **(*c*)** TECs (ccf2390, DI-102, ccf4055, or DI-167) were incubated in suspension on ice for 30 min with 5 μg/ml function blocking integrin α3 subunit antibody (P1B5) or mouse IgG as a control, and then the cells were incubated on a thin-layer of Matrigel in M199 + 5% FBS for 3 hours at 37°C in the presence of 5 μg/ml P1B5 or mouse IgG, followed by cell lysis. Primary antibodies used for immunoblotting were: Anti-phospho-S6 kinase (Cell signaling, #9205, thr389), anti-total-S6 kinase (Cell signaling, #9202), anti-Phospho-Akt (Cell signaling, #9271, Ser473), anti-total-Akt (Cell signaling, #4691, [C67E7]), anti-GAPDH (Santa Cruz biotechnology, Sc-32233, [6C5]), anti-integrin α3 (Proteintech, 21992-1-AP), anti-β-tubulin (Santa Cruz biotechnology, sc-101527), anti-phospho-SGK1 (GeneTex, GTX32413, S422), and anti-total-SGK1 (EMD Millipore, 07-315). Whole cell lysates derived from the same experiment were subject to SDS-PAGE and immunoblotting with the indicated primary antibodies in parallel. Independent experiments were performed at least two times with similar results. Source data are provided as a source data file.

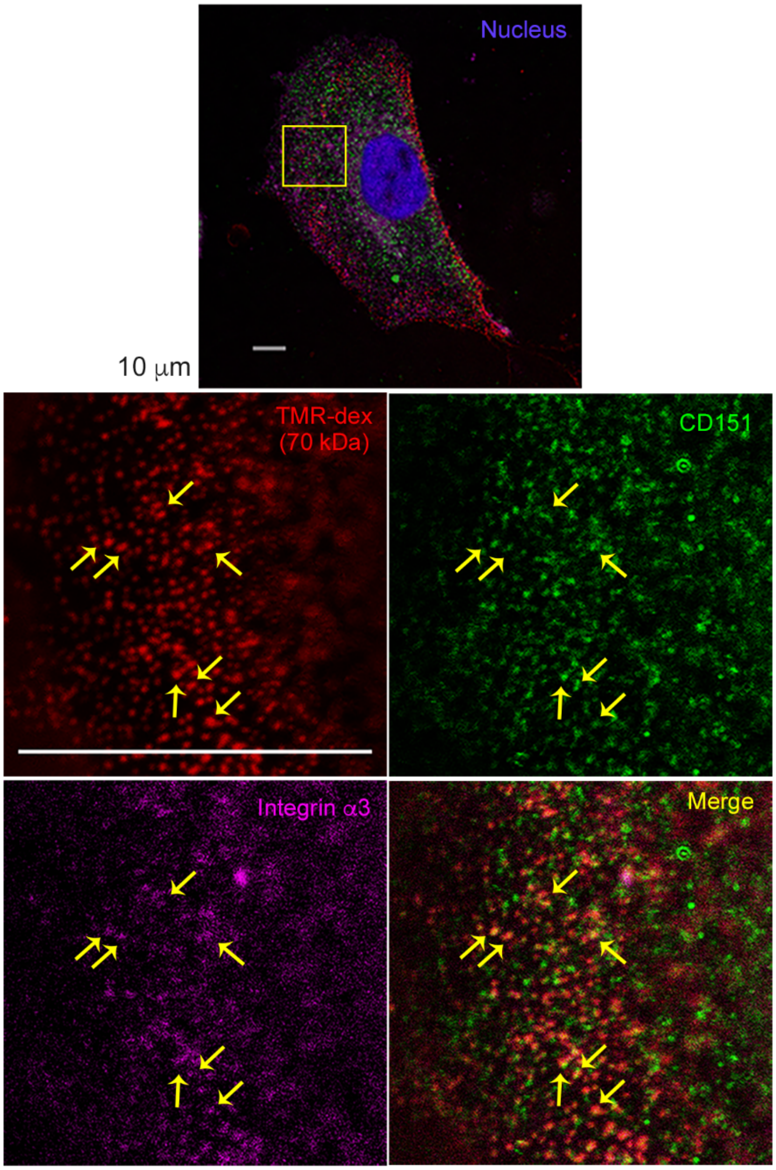

SFig. 13. Integrin α3β1 and CD151 are internalized by macropinocytosis. TEC (ccf2390) adherent to collagen I (10 μg/ml) was pretreated with 10 μM chloroquine for 30 min to maximize observance of intracellular vesicles, and then additionally incubated for 30 min with TMR-dex at 37°C, followed by double-label immunofluorescence for the integrin α3 subunit and CD151. Cells were fixed with 4% paraformaldehyde, permeabilized with 0.3% Triton X-100, blocked (5% horse serum & 5% BSA in TBST), and reacted with mouse anti-integrin α3 subunit antibody (Abcam, ab8985, clone 29A3) and rabbit anti-CD151 antibody (Epitomics, EP6875), followed by Alexa-fluor-488-goat anti-rabbit IgG

(Thermo Fisher Scientific, A11034) and Alexa-fluor-633-goat anti-mouse IgG (A21052) secondary antibodies. Slides were then mounted on VectaShield antifade mounting medium with DAPI. Yellow arrows show colocalization of TMR-dex, CD151, and the integrin α3 subunit. Images were captured by the Leica SP8 confocal microscope. Colocalization of TMR-dex-positive vesicles with CD151-positive vesicles was 87%, based on the Manders' Coefficients (M1=0.908, M2=0.872); and colocalization of TMR-dex-positive vesicles with integrin α3-positive vesicles was 83% (M1=0.831; M2=0.905). Independent experiments were performed at least two times with similar results.

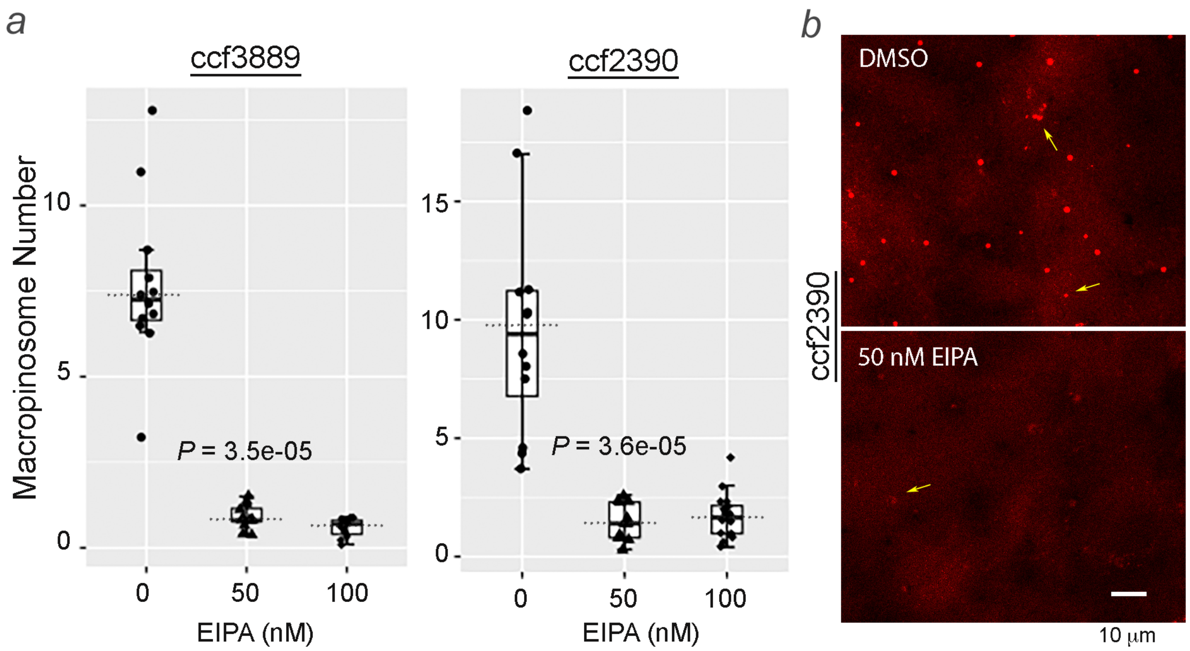

SFig. 14. The Inhibition of macropinocytosis with EIPA at 50 and 100 nM. TECs adherent to collagen I in M199 + 5% FBS with EIPA or vehicle control (DMSO) for 10 min were incubated with 25 μg/ml TMR-dex at 37 °C for 30 min, followed by fixation with 4 % paraformaldehyde. Images were captured using the Leica SP8 confocal microscope. **(*a*)** Macropinocytosis was quantified as macropinosome number (the average number of macropinosmes in a cell per field). Boxes indicate first and third quartiles, bands indicate medians, and whiskers indicate ±1.5 interquartile range. Dotted lines denote means. n = number of different fields: In TEC (ccf2390), n = 12 for DMSO group, n = 12 for 50 nM EIPA, and n = 16 for 100 nM EIPA; In TEC (ccf3889), n = 12 for DMSO group, n = 12 for 50 nM EIPA, and n = 11 for 100 nM EIPA. Statistical analysis: two-sided Wilcoxon rank-sum test between DMSO (vehicle control) and 50 nM EIPA group. Source data are provided as a source data file. **(*b*)** Representative images are shown.

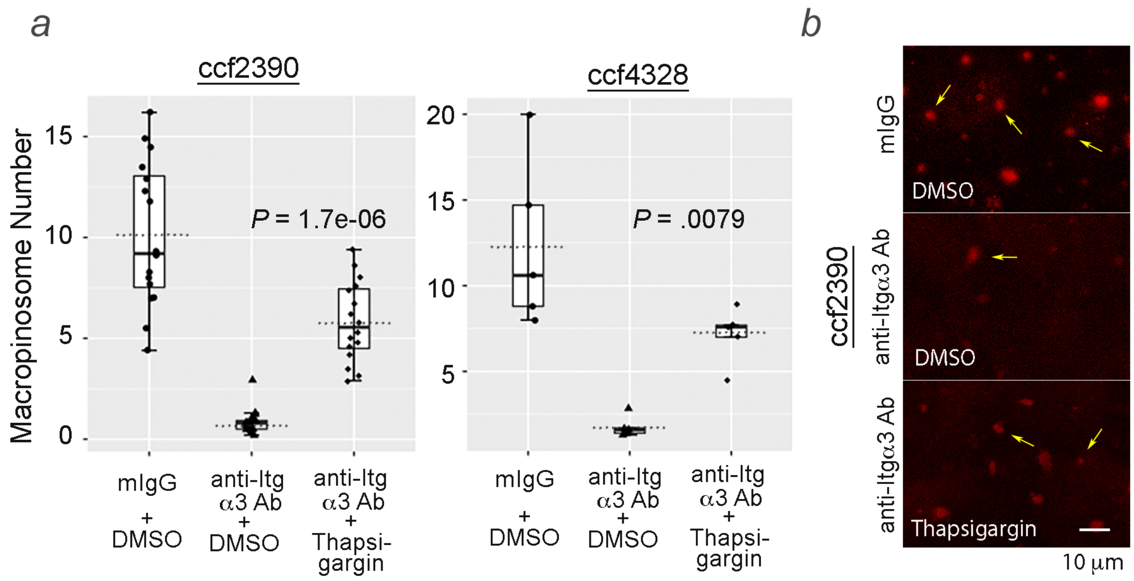

SFig. 15. Thapsigargin, which releases calcium from intracellular calcium storage, partially reverses the inhibition of macropinocytosis observed with the blocking antibody to integrin α3. TECs adherent to collagen I in the presence of mouse IgG (mIgG) or integrin α3 blocking antibody (anti-Itgα3 Ab, P1B5) in M199 + 5% FBS were treated with vehicle control (DMSO) or 1 μM Thapsigargin for 10 min, and then incubated with 25 μg/ml TMR-dex at 37 °C for 30 min, followed by fixation with 4 % paraformaldehyde. Images were captured using the Leica SP8 confocal microscope. **(*a*)** Macropinocytosis was quantified as Macropinosome Number (the average number of macropinosmes in a cell per field). Boxes indicate first and third quartiles, bands indicate medians, and whiskers indicate ±1.5 interquartile range. Dotted lines denote means. n = number of different fields: In TEC (ccf2390), n = 16 for mouse IgG + DMSO condition, n = 16 for anti-Itgα3 Ab + DMSO condition, and n = 16 for anti-Itgα3 Ab + Thapsigargin condition; In TEC (ccf4328), n = 5 for mouse IgG + DMSO condition, n = 5 for anti-Itgα3 Ab + DMSO condition, and n = 5 for anti-Itgα3 Ab + Thapsigargin condition. Statistical analysis: two-sided Wilcoxon rank-sum test between anti-Itgα3 Ab + DMSO condition versus anti-Itgα3 Ab + Thapsigargin condition. Source data are provided as a source data file. **(*b*)** Representative images are shown.

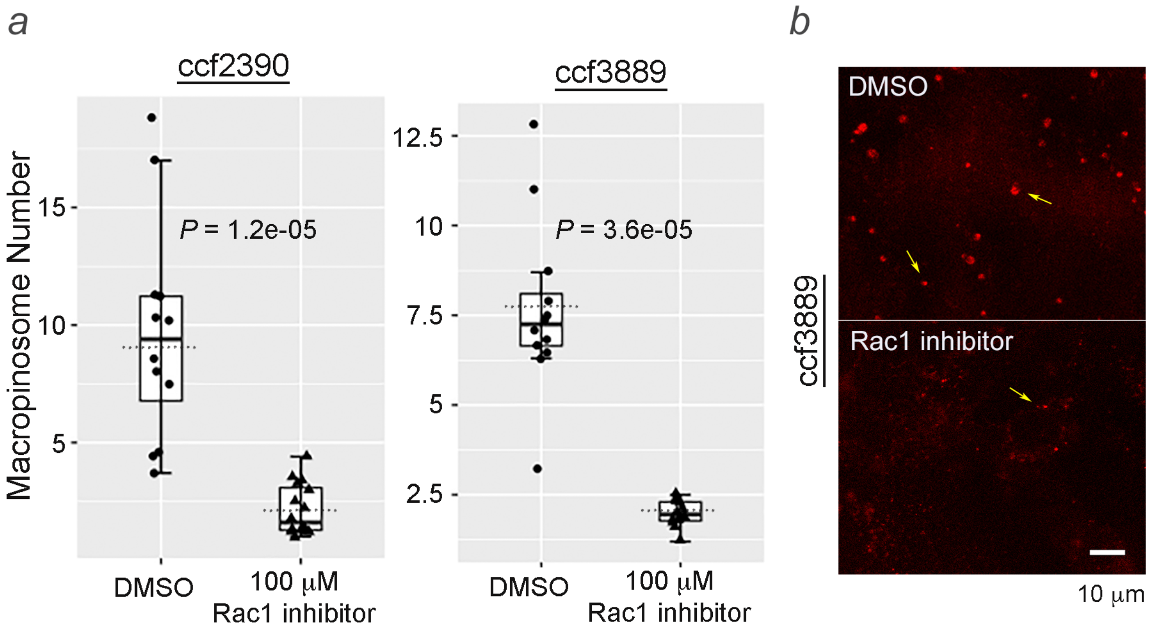

SFig. 16. Inhibition of macropinocytosis with a Rac1 inhibitor, NSC23766. TECs adherent to collagen I in M199 + 5% FBS with 100 μM NSC23766 or vehicle control (DMSO) for 10 min were incubated with 25 μg/ml TMR-dex at 37°C for 30 min, followed by fixation with 4% paraformaldehyde. Images were captured using the Leica SP8 confocal microscope. (***a***) Macropinocytosis was quantified as Macropinosome Number (the average number of macropinosmes in a cell per field). Boxes indicate first and third quartiles, bands indicate medians, and whiskers indicate ±1.5 interquartile range. Dotted lines denote means. n = number of different fields: In TEC (ccf2390), n = 12 for the DMSO condition, and n = 16 for the Rac1 inhibitor condition; In TEC (ccf3889), n = 12 for the DMSO condition and n=12 for the Rac1 inhibitor condition. Statistical analysis: two-sided Wilcoxon rank-sum test. Source data are provided as a source data file. (***b***) Representative images are shown.

Supplementary Table 1
Fresh GBM tumor samples were obtained from the Cleveland Clinic Brain Tumor Bank and the Brain Tumor Bank of the University Hospitals in a coded and de-identifiable manner (denoted as Tumor ID), in accordance with the guidelines and policies of the Cleveland Clinic and the University Hospitals Institutional Review Boards. Samples from newly diagnosed GBM are denoted as nGBM, and samples from recurrent GBM are denoted as rGBM. Tumor-associated endothelial cells (TECs) were isolated from each GBM sample as described in the Methods.

Tumor ID	Diagnosis<br>nGBM: newly diagnosed GBM<br>rGBM: recurrent GBM
ccf2277	nGBM
ccf2352	nGBM
ccf2388	nGBM
ccf2390	nGBM
ccf2405	nGBM
ccf2445	nGBM
ccf2455	nGBM
ccf2566	nGBM
ccf2687	nGBM
ccf3889	nGBM
ccf4055	nGBM
ccf4259	nGBM
ccf4272	nGBM
ccf4328	nGBM
cw850	nGBM
cw880	nGBM
DI-102	nGBM
DI-167	nGBM
DI-247	nGBM
DI-337	nGBM
ccf2515	rGBM
ccf3679	rGBM
ccf4268	rGBM
