# Supplementary material for: Integrin α3β1 promotes vessel formation of glioblastoma-associated endothelial cells through calcium-mediated macropinocytosis and lysosomal exocytosis
Source: Nat Commun. 2022 Jul 25;13:4268. doi: 10.1038/s41467-022-31981-2 (PMC9314429; doi:10.1038/s41467-022-31981-2)
Supplement: Supplementary file 2 — Reporting Summary [file 41467_2022_31981_MOESM2_ESM.pdf]

nature research

Corresponding author(s): Candece L. Gladson, M.D.

Last updated by author(s): April 26, 2022

Reporting Summary

Nature Research wishes to improve the reproducibility of the work that we publish. This form provides structure for consistency and transparency in reporting. For further information on Nature Research policies, see Authors & Referees and the Editorial Policy Checklist.

Statistics

For all statistical analyses, confirm that the following items are present in the figure legend, table legend, main text, or Methods section.

n/a	Confirmed	
☐	☒	The exact sample size (*n*) for each experimental group/condition, given as a discrete number and unit of measurement
☐	☒	A statement on whether measurements were taken from distinct samples or whether the same sample was measured repeatedly
☐	☒	The statistical test(s) used AND whether they are one- or two-sided<br>*Only common tests should be described solely by name; describe more complex techniques in the Methods section.*
☒	☐	A description of all covariates tested
☒	☐	A description of any assumptions or corrections, such as tests of normality and adjustment for multiple comparisons
☐	☒	A full description of the statistical parameters including central tendency (e.g. means) or other basic estimates (e.g. regression coefficient) AND variation (e.g. standard deviation) or associated estimates of uncertainty (e.g. confidence intervals)
☐	☒	For null hypothesis testing, the test statistic (e.g. *F*, *t*, *r*) with confidence intervals, effect sizes, degrees of freedom and *P* value noted<br>*Give P values as exact values whenever suitable.*
☒	☐	For Bayesian analysis, information on the choice of priors and Markov chain Monte Carlo settings
☒	☐	For hierarchical and complex designs, identification of the appropriate level for tests and full reporting of outcomes
☒	☐	Estimates of effect sizes (e.g. Cohen's *d*, Pearson's *r*), indicating how they were calculated

Our web collection on statistics for biologists contains articles on many of the points above.

Software and code

Policy information about availability of computer code

Data collection	A Leica DM 5500 upright fluorescence microscope equipped with a Leica DFC 7000T camera, a Leica DMI 6000 inverted fluorescence microscope equipped with a Hamamatsu ORCA Flash 4 camera and an inverted Leica SP8 confocal microscope were run by Leica LASX software; IncuCyte Zoom (Essen Bioscience) for imaging in Fig5h ; Captured images were viewed by Adobe Photoshop CC; Gen5 (version 3.00.19, BioTeck Instruments) for calcium influx assay in Fig6g and Fig6h
Data analysis	ImageJ (1.51s) for image analysis in Fig2f, Fig2h, Fig2i, Fig4c, Fig5a, Fig5b, Fig5d, Fig5e, Fig5f, Fig6e, Fig6f and Fig6g; DAVID Bioinformatics Resources 6.8(https://david.ncifcrf.gov/summary.jsp) for gene enrichment analysis in Fig2a and Fig2c; EASE version 2.0 software for microarray data mentioned in Fig2 and sFig3; Velocity 6.3 (PerkinElmer) for FIg6c and Fig6d; R (http://www.R-project.org) for statistical analysis; GraphPad Prism for Fig1, Fig2f, Fig2h, Fig3b, and Fig3d.

For manuscripts utilizing custom algorithms or software that are central to the research but not yet described in published literature, software must be made available to editors/reviewers. We strongly encourage code deposition in a community repository (e.g. GitHub). See the Nature Research guidelines for submitting code & software for further information.

Data

Policy information about availability of data

All manuscripts must include a data availability statement. This statement should provide the following information, where applicable:

- Accession codes, unique identifiers, or web links for publicly available datasets
- A list of figures that have associated raw data
- A description of any restrictions on data availability

Data Availability: The gene expression microarray data have been deposited in the GEO public database (GSE137902), and are publicly available. To review GEO accession GSE137902: Go to https://www.ncbi.nlm.nih.gov/geo/query/acc.cgi?acc=GSE137902
Source data underlying figures (1a-d, 2a-f, 2h & I, 3a-d, 4c, 5a-f, and 6e-j) and supplementary figures (2b, 3a&b, 4, 6b, 7b, 8b, 10, 12a-c, 14a, 15a, and 16a) are provided as Source Data files in the Supplemental Information.

Field-specific reporting

Please select the one below that is the best fit for your research. If you are not sure, read the appropriate sections before making your selection.

☒ Life sciences ☐ Behavioural & social sciences ☐ Ecological, evolutionary & environmental sciences

For a reference copy of the document with all sections, see nature.com/documents/nr-reporting-summary-flat.pdf

Life sciences study design

All studies must disclose on these points even when the disclosure is negative.

Sample size	Figure 1a-d (tube formation assays): Sample size was determined based on (i) our prior experience performing tube formation assays that indicated the need for at least 6 replicate assessments of tube and sprout formation for each condition to minimize the effect of variability; (ii) on a pilot study assessing tube and sprout formation in the TECs; and (iii) on the limited number of primary TECs that were available. The sample size utilized was sufficient, as we had a highly significant difference in the number of tubes and sprouts when comparing the TECs and the NECs.<br><br>For Figure2i, Logistically it was feasible for our experiment to include 7 mice total. Thus a power calculation was performed to ensure that this sample size would result in a well powered study. Each mouse contributed a normal brain and a GBM tumor sample, so seven mice in total contributed 7 normal brains and 7 GBM tumor samples with a correlation estimate of 0.1. We desired to quantify the mean fluorescence intensity of integrin α3 staining on CD31-positive pixels or the mean fluorescence intensity of CD151 staining on CD31-positive pixels of at least 10 percentage points. Based on pilot data, we assumed a standard deviation of 10 percentage points. All estimates were inflated by 20% and deflated by 20% to check the sensitivity of the study power to proposed estimates. All results showed a study power of at least 90%.<br><br>Figure 3a-d (tube formation assays): Sample size was determined based on (i) our prior experience performing tube formation assays that indicated the need for at least 6 replicate assessments of tube and sprout formation for each condition to minimize the effect of variability; and (ii) on a pilot study assessing individually the effects of anti-integrin α3 antibody and the effects of anti-integrin α6 antibody on tube and sprout formation. The sample size utilized was sufficient, as we found a highly significant difference in the number of tubes and sprouts when the TECs were treated with the anti-integrin α3 antibody as compared to treatment with mouse IgG; and we found a highly significant difference in the number of tubes when the NECs were treated with the anti-integrin α6 antibody as compared to treatment with rat IgG.<br><br>For Figure 4, we did not do a power analysis as this experiment was totally dependent on the number of fresh GBM tumor samples of adequate size that were available from the Brain Tumor Bank in the time frame of these experiments.<br><br>Figure 5a-f (macropinocytosis assays): Sample size was determined based on (i) our prior experience performing macropinocytosis assays that indicated the need for at least 8 replicate assessments of macropinosome quantification for each condition to minimize the effect of variability; (ii) on a pilot study assessing the effects individually of anti-anti-integrin α3 antibody and anti-CD151 antibody on macropinocytosis in the TECs; and (iii) on a pilot study assessing the effect(s) of siRNA on macropinocytosis in the TECs. The sample size utilized was sufficient, as we had a highly significant difference in the macropinosome number or in the macropinocytotic index when comparing the TECs with the NECs. Also, we had highly significant differences in the macropinosome number in TECs when comparing the effect of EIPA versus vehicle DMSO, when comparing the effect of anti-integrin α3 antibody versus mouse IgG, when comparing the effect of anti-CD151 antibody versus mouse IgG, when comparing the effect of siRNA toward integrin α3 versus control siRNA, and when comparing the effect of siRNA toward CD151 versus control siRNA.<br><br>Figure 6e and f (Quantitation of exocytosis using the area of co-localization of TMR-dex with the LAMP1-H4A3 antibody): The sample size was determined based on a pilot study determining that at least 10 replicate assessments of co-localization were required to minimize the effect of variability on quantitating co-localization of TMR-dex with the LAMP1-H4A3 antibody. The sample size utilized was sufficient, as highly significant differences were found in the TECs when assessing co-localization of TMR-dex and LAMP1-H4A3 antibody in the presence of EGTA versus vehicle; and highly significant differences were found in the TECS when assessing co-localization of TMR-dex and LAMP1-H4A3 antibody in the presence of anti-integrin α3 antibody versus mouse IgG.<br><br>Figure 6g and h (Quantitation of macropinocytosis and tube formation): The sample size was determined as above for Figure 5 (macropinocytosis) and Figure 3 (tube formation). The sample size utilized was sufficient, as highly significant differences were found in the TECs when assessing the effect of EGTA versus vehicle control on macropinocytosis, and when assessing the effect of EGTA versus vehicle control on tube formation.<br><br>Figure 6i and j (Quantitation of calcium influx): The sample size was determined based on a pilot study determining that three replicates were necessary for each time point to accurately quantitate calcium influx in the TECs. The sample size utilized was sufficient, as significant differences were found when assessing the effect of anti-integrin α3 antibody versus mouse IgG on calcium influx.
Data exclusions	No data were excluded from the analysis.
Replication	Reproducibility was assessed by replicas and repeated experiments. All experiments were performed at least two times with similar results.
Randomization	Randomization was not performed and was not appropriate. We did not utilize randomization for the animal experiment in Figure 2i, as the mice were control mice in whom tumors spontaneously developed.
Blinding	The same criteria were applied irrespective of sample groups for imaging and experiments. True blinding would require that one had absolutely no knowledge of the group conditions during data collection and analysis, and this is not realistically feasible during data collection and analysis. For example, for some experiments one needs to set a threshold that applies to both positive and negative controls, and this

requires knowledge of positive and negative control groups. Every effort was made to carry out data collection and analysis in a manner that was independent of the group or condition within an experiment.

Reporting for specific materials, systems and methods

We require information from authors about some types of materials, experimental systems and methods used in many studies. Here, indicate whether each material, system or method listed is relevant to your study. If you are not sure if a list item applies to your research, read the appropriate section before selecting a response.

Materials & experimental systems

n/a	Involved in the study
☐	☒ Antibodies
☒	☐ Eukaryotic cell lines
☒	☐ Palaeontology
☐	☒ Animals and other organisms
☐	☒ Human research participants
☒	☐ Clinical data

Methods

n/a	Involved in the study
☒	☐ ChIP-seq
☒	☐ Flow cytometry
☒	☐ MRI-based neuroimaging

Antibodies

Antibodies used

The primary antibodies used for immunofluorescence analysis of human cells and tissues were purchased as follows: Rabbit anti-CD31 antibody (NBP1-71663) from Novus Biologicals; mouse monoclonal anti-integrin alpha3 subunit (MAB1952z, clone P1B5); rabbit anti-vwf (AB7356) from Millipore; rabbit anti-VE-Cadherin antibody (ab33168), rabbit anti-integrin alpha3 subunit (ab131055), rabbit anti-CD151 (ab201174), mouse anti-vwf (ab68545), rabbit anti-SNX5 antibody (ab180520, clone EPR14368) and rabbit anti-LAMP1 antibody (ab24170) from Abcam; rabbit anti-integrin alpha6 subunit (T0919) from Epitomics; goat anti-laminin alpha5 IgG (sc-16592) from Santa Cruz and mouse anti-LAMP1 antibody (H4A3) from the Developmental Studies Hybridoma Bank. Secondary antibodies were: Alexa-Fluor-594-conjugated goat anti-mouse IgG (A11032), Alexa-Fluor-594-conjugated donkey anti-goat IgG (A11058), Alexa-Fluor-594-conjugated goat anti-rabbit IgG (A11037) and Alexa-Fluor-488-conjugated goat anti-mouse IgG (A21121) from Thermo Fisher Scientific; and Alexa-Fluor-488-conjugated goat anti-rabbit IgG (A11034) from Life Technologies. For immunofluoresence analysis of mouse glioma, the primary antibodies used were rabbit anti-integrin alpha3 subunit (Millipore-Sigma, AB1920), rabbit anti-integrin alpha6 subunit (Bioss, BS-2641R), rabbit anti-CD151 (Epitomics, 5901-1, clone EP6875) and rat anti-mouse CD31 (Dianova, DIA-310) and the secondary antibodies were Alexa-Fluor-568-conjugated goat anti-rabbit IgG (A11036) and Alexa-Fluor-488-conjugated goat anti-rat IgG (A21208) from ThermoFisher Scientific.

The primary antibodies used were: Anti-integrin alpha6 subunit (T0919) and anti-CD151 (EP6875) from Epitomics Inc., anti-integrin alpha3 subunit (21992-1-AP) and anti-integrin beta1 subunit (12594-1-AP) from Proteintech; anti-integrin alphaV subunit (4711P) from Cell Signaling; anti-integrin beta3 subunit (ab7167, clone BV4) and anti-VE-cadherin (ab33168) from Abcam; anti-glyceraldehyde-3-phosphate dehydrogenase (GAPDH, sc-365062, clone 6C5), and anti-beta-tubulin (sc-101527) from Santa Cruz Biotechnology. The peroxidase-conjugated secondary antibodies (NA9340V and NA931V) were purchased from GE Healthcare.

Fig. 1f
Goat anti-laminin α5 (Santa Cruz sc-16592, 1:200) followed by donkey anti-goat IgG Alexa Fluor 594 (Thermo Fisher Scientific A11058, 1:200).

Fig. 2e Western blot
Lab notebook (Western blot binder): eb2012_0803 and other dates
Anti-Integrin alpha3 (Proteintech, 21992-1-AP, 1:1,000)
Anti-Integrin alpha6 (Epitomics, T0919, 1:50)
Anti-Integrin alphaV (Cell Signaling Technology, 4911P, 1:1,200)
Anti-Integrin beta1 (Proteintech, 12594-1-AP, 1:250)
Anti-Integrin beta3 (BV4, Abcam, ab7167, 1:1000)
Anti-CD151 (Epitomics, 5901-1, clone EP6875, 1:1,000)
Anti-VE-Cadherin (Abcam ab33168, 1:700)
Anti-GAPDH (Santa Cruz biotechnology, Sc-32233, [6C5], 1:2,000)

Fig. 2f
Mouse anti-integrin α3 (Milipore P1B5, 1:100) followed by goat anti-mouse IgG Alexa Fluor 594 (Thermo Fisher Scientific A11032, 1:100).

Fig. 2h Human GBM tissue arrays
Rabbit anti-integrin α6 (Bioss BS-2641R, 1:200) followed by goat anti-rabbit IgG Alexa Fluor 568 (Thermo Fisher Scientific A11037, 1:200). Rabbit anti-CD151 (Epitomics, 5901-1, clone EP6875, 1:300) followed by goat anti-rabbit IgG Alexa Fluor 568 (Thermo Fisher Scientific A11037, 1:200). Rabbit anti-integrin α3 (Millipore-Sigma, AB1920, 1:500) followed by goat anti-rabbit IgG Alexa Fluor 568 (Thermo Fisher Scientific A11037, 1:200).

Fig. 2g Human GBM sections
Rabbit anti-integrin alpha3 (Abcam, ab131055, 1:70) followed by goat anti-rabbit IgG Alexa Fluor 594 (Thermo Fisher Scientific A11037, 1:200). Mouse anti-vWF antibody (F8/86, ThermoFisher Scientific MA5-14029, 1:50) followed by goat anti-rabbit IgG Alexa Fluor 488 (Thermo Fisher Scientific, A11034, 1:200).

Fig. 2i Mouse sections
Rabbit anti-integrin α6 (Bioss BS-2641R, 1:200) followed by goat anti-rabbit IgG Alexa Fluor 568 (Thermo Fisher Scientific A11037, 1:200). Rabbit anti-CD151 (Epitomics, 5901-1, clone EP6875, 1:300) followed by goat anti-rabbit IgG Alexa Fluor 568 (Thermo Fisher Scientific A11037, 1:200). Rabbit anti-integrin α3 (Millipore-Sigma, AB1920, 1:500) followed by goat anti-rabbit IgG Alexa Fluor 568 (Thermo Fisher Scientific A11037, 1:200).

Fig. 4b
Rabbit anti-CD31 (Novus Biologicals NBP1-71663, 1:75) followed by goat anti-rabbit IgG Alexa Fluor 488 (Thermo Fisher Scientific A11034, 1:200).

Fig. 5f
Anti-integrin alpha3 (Proteintech, 21992-1-AP, 1:1,000)
Anti-CD151 (Abcam, ab131028, 1:1,000)
Anti-b-tubulin (Santa-Cruz, sc101527, 1:2,000)

Fig. 6a
Mouse anti-lamp1 (Developmental Studies Hybridoma Bank H4A3, 1:100 or 2ug/ml) followed by goat anti-mouse IgG Alexa Fluor 488 (Thermo Fisher Scientific A21121, 1:200).

Fig. 6cd
Wheat Germ Agglutinin (WGA) Alexa Fluor 647 Conjugate (Thermo Fisher Scientific W32466, 5ug/ml final concentration). Mouse anti-lamp1 (Developmental Studies Hybridoma Bank H4A3, 1:100 or 2ug/ml) followed by goat anti-mouse IgG Alexa Fluor 488 (Thermo Fisher Scientific A21121, 1:200).

Fig. 6ef
Mouse anti-lamp1 (Developmental Studies Hybridoma Bank H4A3, 1:100 or 2ug/ml) followed by goat anti-mouse IgG Alexa Fluor 488 (Thermo Fisher Scientific A21121, 1:200).

SFig. 5
Rabbit anti-VE-cadherin (Abcam, ab33168, 1:100) followed by goat anti-rabbit IgG Alexa Fluor 488 (Thermo Fisher Scientific, A11034, 1:100).

SFig. 6
Rabbit anti-integrin alpha3 (Abcam, ab131055, 1:70) followed by goat anti-rabbit IgG Alexa Fluor 594 (Thermo Fisher Scientific A11037, 1:200). Mouse anti-vWF antibody (F8/86, ThermoFisher Scientific MA5-14029, 1:50) followed by goat anti-rabbit IgG Alexa Fluor 488 (Thermo Fisher Scientific, A11034, 1:200).

SFig. 7
Function blocking antibody towards the integrin alpha7 subunit (9.1 ITGA7, Developmental Studies Hybridoma Bank-DSHB, 5ug/ml or 10ug/ml) or control IgG (mouse IgG, SC-2025, Santa Cruz Biotechnology, Inc., 5ug/ml or 10ug/ml).

SFig. 9
Rabbit anti-SNX5 (EPR14358, Abcam ab180520, 1:100) followed by goat anti-rabbit IgG Alexa Fluor 488 (Thermo Fisher Scientific, A11034, 1:100).

SFig. 12
Anti-Phospho-S6 Kinase (Cell signaling, #9205, thr389, 1:1,000)
Anti-total-S6 Kinase (Cell signaling, #9202, 1:1,000)
Anti-Phospho-Akt (Cell signaling, #9271, Ser473, 1:1,000)
Anti-total-Akt (Cell signaling, #4691, [C67E7], 1:1,000)
Anti-GAPDH (Santa Cruz biotechnology, Sc-32233, [6C5], 1:1,500 or 1:2,000)
Anti-Integrin Alpha3 (Proteintech, 21992-1-AP, 1:1,000)
Anti-beta-Tubulin (Santa Cruz biotechnology, sc-101527, 1:2,000)
Anti-phospho-SGK1 (GeneTex, GTX32413, S422, 1:1,000)
Anti-total-SGK1 (EMD Millipore, 07-315, 1:2,000)

SFig. 13

Rabbit anti-CD151 (Epitomics, EP6875, 1:200) followed by goat anti-rabbit IgG Alexa Fluor 488 (LifA11034, 1:200). Mouse anti-integrin alpha3 subunit (Abcam, ab8985, clone 29A3, 1:200) followed by goat anti-mouse IgG Alexa Fluor 633 (Thermo Fisher Scientific, A21052, 1:200).

Validation

Western blot (WB), flow cytometry (Flow), immunofluorescence (IF), immunohistochemistry(IHC), immunohistochemistry on paraffin section (IHC-P), Function blocking (FUNC), Enzyme-linked immunosorbent assay (ELISA), and Immunoprecipitation (IP).

Rabbit anti-CD31 antibody (NBP1-71663) from Novus Biologicals
https://www.novusbio.com/products/cd31-pecam-1-antibody_nbp1-71663
Validated for WB, Flow, IF, IHC and IHC-P

Mouse monoclonal anti-integrin α3 subunit (MAB1952z, clone P1B5) from Millipore
https://www.emdmillipore.com/US/en/product/Anti-Integrin-3-Antibody-clone-P1B5-azide-free,MM_NF-MAB1952Z
Validated for IF, IHC and FUNC

Rabbit anti-vwf (AB7356) from Millipore
http://www.emdmillipore.com/US/en/product/Anti-von-Willebrand-Factor-Antibody,MM_NF-AB7356
Validated for IHC, IHC-P and ELISA

Mouse anti-vwf antibody (F8/86, MA5-14029) from ThermoFisher Scientific
https://www.thermofisher.com/antibody/product/VWF-Antibody-clone-F8-86-Monoclonal/MA5-14029
Validated for WB, Flow, IF, IHC and IHC-P

Rabbit anti-VE-Cadherin antibody (ab33168) from Abcam
https://www.abcam.com/ve-cadherin-antibody-intercellular-junction-marker-ab33168.html
Validated for WB and IF

Rabbit anti-integrin α3 subunit (ab131055) from Abcam
https://www.abcam.com/integrin-alpha-3-antibody-ab131055.html
Validated for WB, IF, IHC and IHC-P

Rabbit anti-CD151 (ab201174) from Abcam
https://www.abcam.com/cd151-antibody-ab201174.html
Validated for WB and IHC-P

Mouse anti-vwf (ab68545) from Abcam
https://www.abcam.com/von-willebrand-factor-antibody-21-43-ab68545.html
Validated for WB and IHC-P

Rabbit anti-SNX5 antibody (ab180520, clone EPR14368) from Abcam
https://www.abcam.com/snx5-antibody-epr14358-ab180520.html
Validated for WB, Flow, IF, and IHC-P

Rabbit anti-LAMP1 antibody (ab24170) from Abcam
https://www.abcam.com/lamp1-antibody-lysosome-marker-ab24170.html
Validated for WB, and IHC-P

Rabbit anti-integrin α6 subunit (T0919) from Epitomics has been purchased by Abcam (ab75737)
https://www.abcam.com/integrin-alpha-6-antibody-ab75737.html
Validated for WB, IF, and IHC-P

Goat anti-laminin α5 IgG (sc-16592) from Santa Cruz
http://datasheets.scbt.com/sc-16592.pdf
Validated for WB, IP, IF, and ELISA

Mouse anti-LAMP1 antibody (H4A3) from the Developmental Studies Hybridoma Bank
https://dshb.biology.uiowa.edu/H4A3
Validated for WB, IF and IHC

Mouse anti-integrin 7 antibody (9.1ITGA7) from the Developmental Studies Hybridoma Bank
https://dshb.biology.uiowa.edu/9-1-ITGA7
Validated for WB, Flow, IF, IP, and FUNC

Rabbit anti-integrin α3 subunit (Millipore-Sigma, AB1920)
http://www.emdmillipore.com/US/en/product/Anti-Integrin-3-Antibody,MM_NF-AB1920
Validated for WB, IF, IP, and ELISA

Rabbit anti-integrin α6 subunit (Bioss, BS-2641R)
https://www.biossusa.com/products/bs-2641r
Validated for WB, IF, IHC, IHC-P, and ELISA

Rabbit anti-CD151 (Epitomics, 5901-1, clone EP6875) has been purchased by Abcam (ab131028)
https://www.abcam.com/cd151-antibody-ep6875-ab131028.html
Validated for WB, FLOW, and IHC-P

Rat anti-mouse CD31 (Dianova, DIA-310)
https://www.dianova.com/en/produkte/dia-310-anti-cd31-ms-from-rat-clone-sz31-for-mouse-ffpe-tissue-500-%C2%B5l/
Validated for WB, IF, and IHC-P

Rabbit anti-phospho-S6 kinase (Cell signaling, #9205, thr389)
https://www.cellsignal.com/products/primary-antibodies/phospho-p70-s6-kinase-thr389-antibody/9205
Validated for WB

Rabbit anti-total-S6 kinase (Cell signaling, #9202)
https://www.cellsignal.com/products/primary-antibodies/p70-s6-kinase-antibody/9202
Validated for WB and IP

Rabbit anti-Phospho-Akt (Cell signaling, #9271, Ser473)
https://www.cellsignal.com/products/primary-antibodies/phospho-akt-ser473-antibody/9271
Validated for WB, IP, IF, and FLOW

Rabbit anti-total-Akt (Cell signaling, #4691, [C67E7])
https://www.cellsignal.com/products/primary-antibodies/akt-pan-c67e7-rabbit-mab/4691
Validated for WB, IP, IF, IHC-P, and FLOW

Mouse anti-GAPDH (Santa Cruz biotechnology, Sc-32233, [6C5])
https://datasheets.scbt.com/sc-32233.pdf
Validated for WB, IP, and IF

Rabbit anti-integrin a3 (Proteintech, 21992-1-AP)
https://www.ptglab.com/products/ITGA3-Antibody-21992-1-AP.htm
Validated for WB, IP, FLOW, IF, and ELISA

Mouse anti-b-tubulin (Santa Cruz biotechnology, sc-101527)
https://datasheets.scbt.com/sc-101527.pdf
Validated for WB, IP, IHC-P, and ELISA

Rabbit anti-phospho-SGK1 (GeneTex, GTX32413, S422)
https://antibodypedia.com/gene/19734/SGK1/antibody/2977965/GTX32413
Validated for WB, IF, and IHC

Rabbit anti-total-SGK1 (EMD Millipore, 07-315)
https://www.emdmillipore.com/US/en/product/Anti-SGK1-Antibody,MM_NF-07-315
Validated for WB, and IP

Alexa-Fluor-568-conjugated goat anti-rabbit IgG (A11036) from ThermoFisher Scientific
https://www.thermofisher.com/antibody/product/Goat-anti-Rabbit-IgG-H-L-Highly-Cross-Adsorbed-Secondary-Antibody-Polyclonal/A-11036

Alexa-Fluor-488-conjugated goat anti-rat IgG (A21208) from ThermoFisher Scientific
https://www.thermofisher.com/antibody/product/Donkey-anti-Rat-IgG-H-L-Highly-Cross-Adsorbed-Secondary-Antibody-Polyclonal/A-21208

Alexa-Fluor-594-conjugated goat anti-mouse IgG (A11032) from Thermo Fisher Scientific
https://www.thermofisher.com/antibody/product/Goat-anti-Mouse-IgG-H-L-Highly-Cross-Adsorbed-Secondary-Antibody-Polyclonal/A-11032

Alexa-Fluor-594-conjugated donkey anti-goat IgG (A11058) from Thermo Fisher Scientific
https://www.thermofisher.com/antibody/product/Donkey-anti-Goat-IgG-H-L-Cross-Adsorbed-Secondary-Antibody-Polyclonal/A-11058

Alexa-Fluor-594-conjugated goat anti-rabbit IgG (A11037) from Thermo Fisher Scientific
https://www.thermofisher.com/antibody/product/Goat-anti-Rabbit-IgG-H-L-Highly-Cross-Adsorbed-Secondary-Antibody-Polyclonal/A-11037

Alexa-Fluor-488-conjugated goat anti-mouse IgG (A21121) from Thermo Fisher Scientific
https://www.thermofisher.com/antibody/product/Goat-anti-Mouse-IgG1-Cross-Adsorbed-Secondary-Antibody-Polyclonal/A-21121

Alexa Fluor 488-conjugated goat anti-mouse IgG (A32723) from Life Technologies
https://www.thermofisher.com/antibody/product/Goat-anti-Mouse-IgG-H-L-Highly-Cross-Adsorbed-Secondary-Antibody-Polyclonal/A32723

Alexa-Fluor-488-conjugated goat anti-rabbit IgG (A11034) from Life Technologies (Thermo Fisher Scientific)
https://www.thermofisher.com/antibody/product/Goat-anti-Rabbit-IgG-H-L-Highly-Cross-Adsorbed-Secondary-Antibody-Polyclonal/A-11034

Animals and other organisms

Policy information about studies involving animals; ARRIVE guidelines recommended for reporting animal research

Laboratory animals	Nine to ten week-old Nestin-tva, lnk4a-arf -/-transgenic mice with a C57BL/6 background were injected intracranially with vector-infected chicken fibroblasts (DF-1) producing RCAS-PDGF-B virus35 in accordance with the guidelines and policies regarding animal use at Emory University (IACUC approval # 2003253). The housing conditions for the mice were as follows: 12 h light/12 h darkness; temperature was 72 degrees Fahrenheit; and humidity was 40-50%.<br><br>Tumor sections from 5 female and 2 male mice were used for the experiment described in Figure 2i.
Wild animals	The study did not involve wild animals.
Field-collected samples	The study did not involve samples collected in the fields.
Ethics oversight	The animal protocol was approved by the Emory University Institutional Animal Care and Use Committee (IACUC).

Note that full information on the approval of the study protocol must also be provided in the manuscript.

Human research participants

Policy information about studies involving human research participants

Population characteristics	For IRB #2559 from the Cleveland Clinic: Patients of both genders and of all ethnic backgrounds that were 18 years in age or older, and with a diagnosis of glioblastoma tumor (newly-diagnosed or recurrent). The patients were required to be 18 years of age or older, as glioma tumors in the pediatric population are very different from adult glioma or glioblastoma tumors.<br><br>For IRB #12-1010: CASE 9312 BBTC Tissue Registry from the Cleveland Clinic: Patients of both genders and of all ethnic backgrounds that were 18 years in age or older, and with a diagnosis of glioblastoma tumor (newly-diagnosed or recurrent).<br><br>For IRB # 4Y02, 10Z07 and 1307 from the University Hospitals:<br>Patients of both genders and of all ethnic backgrounds that had a diagnosis of newly-diagnosed glioblastoma tumor. The patients were required to be 21 years of age or older, as glioma tumors in the pediatric population are very different from adult glioma or glioblastoma tumors.<br><br>For IRB #14-1427 from the Cleveland Clinic: For the glioblastoma biopsy sections, patients of both genders and of all ethnic backgrounds that were 18 years in age or older, and with a diagnosis of newly-diagnosed glioblastoma tumor. For the normal brain sections, patients of both genders and of all ethnic backgrounds that were 18 years in age or older and with a diagnosis of normal brain at autopsy.
Recruitment	For IRB #2559 from the Cleveland Clinic: All patients with a diagnosis of glioblastoma that were 18 years of age or older and with the capacity to provide consent were recruited.<br><br>For IRB #12-1010: CASE 9312 BBTC Registry from the Cleveland Clinic: All patients with a diagnosis of glioblastoma that were 18 years of age or older were recruited.<br><br>For IRB #4Y02, 10Z07 and 1307 from the University Hospitals: All patients greater than 21 years of age with a diagnosis of newly diagnosed glioblastoma who were competent to consent were recruited.<br><br>For IRB #14-1427 from the Cleveland Clinic: For the glioblastoma biopsy sections, all paraffin blocks with a diagnosis of newly-diagnosed glioblastoma that were from patients 18 years of age or older were recruited. For the normal brain sections, all paraffin blocks with a diagnosis of normal brain at autopsy that were from patients 18 years of age or older were recruited.
Ethics oversight	For IRB #2559 from the Cleveland Clinic: The study protocol was reviewed and approved by the Cleveland Clinic Institutional Review Board (IRB).<br><br>For IRB #12-1010: CASE 9312 BBTC Tissue Registry from the Cleveland Clinic: The study protocol was reviewed and approved by the Cleveland Clinic Institutional Review Board (IRB).<br><br>For IRB #4Y02, 10Z07 and 1307 from the University Hospitals: The study protocol was reviewed and approved by the Protocol Review and Monitoring Committee and the Institutional Review Board at University Hospitals.<br><br>For IRB #14-1427 from the Cleveland Clinic: The study protocol was reviewed and approved by the Cleveland Clinic Institutional Review Board.

Note that full information on the approval of the study protocol must also be provided in the manuscript.
